# Structural and dynamical consistency in lipid vesicles across all-atom, MARTINI, and dissipative particle dynamics

**DOI:** 10.1039/d6ra06719k

**Published:** 2026-09-08

**Authors:** Wandi Xu, Xingfei Wei, Gene Chong, Udaya Dahal, Qiang Cui, Rigoberto Hernandez

**Affiliations:** a Department of Chemistry, Johns Hopkins University Baltimore MD 21218 USA r.hernandez@jhu.edu; b Department of Chemistry, Boston University Boston Massachusetts 02215 USA qiangcui@bu.edu; c Department of Chemical & Biomolecular Engineering, Johns Hopkins University Baltimore MD 21218 USA; d Department of Materials Science and Engineering, Johns Hopkins University Baltimore MD 21218 USA

## Abstract

We develop and validate a mesoscale dissipative particle dynamics (DPD)-4 lipid model, parameterized from an established MARTINI force field. We benchmark this DPD model and a MARTINI model for coarse-grained (CG) lipid vesicles against All-Atom simulations to evaluate their ability to capture structural properties across their respective scales. Dynamical properties are evaluated through comparison between the CG models themselves and against experimental trends. We find that the CG models can capture multiscale spatio-temporal properties of ultrasmall unilamellar vesicles (USUVs) and small unilamellar vesicles (SUVs) with diameters under and over 50 nm, respectively. Specifically, MARTINI and mesoscale DPD lipid models are in agreement in capturing local structural changes of USUVs, such as curvature-dependent lipid packing. For this particular system, structural consistency has been validated using all-atom simulations. At shorter times, dynamical consistency has been validated through agreement between the MARTINI and DPD models. The CG dynamics (with short timescales matched to all-atom simulations) of 60 nm SUVs have been seen to follow the same stretched-exponential relaxation behavior observed in corresponding experiments. This near multi-scale validation highlights a stronger requirement for assessing CG models across length and time. In so doing, we have also demonstrated the potential for using both MARTINI and DPD models to extend vesicle simulations and provide dynamical information in mesoscale regimes with significant computational savings.

## Abstract 2

Dissipative particle dynamics and MARTINI coarse-grained models are benchmarked against all-atom simulations, showing structural and dynamical consistency across scales for lipid vesicles from ultrasmall to small unilamellar sizes.

## Graphical abstract

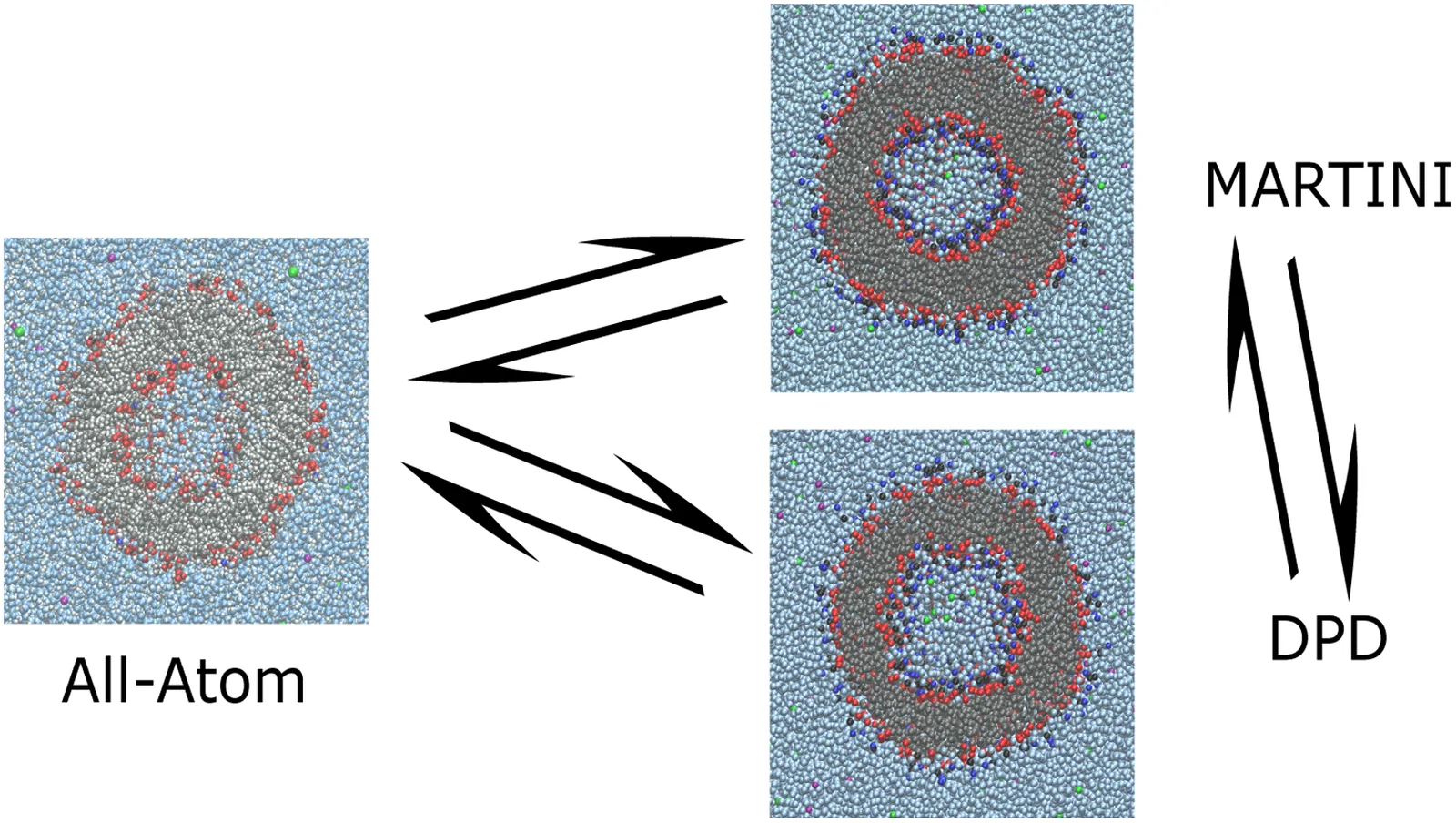

## 1 Introduction

In the ever increasingly-large complex chemical systems whose nonequilibrium behavior needs to be characterized while retaining the fundamental nature of molecular interactions, the need for multi-scale—often CG—representations that offer both thermodynamic and dynamical consistency has become ever pressing.^1,2^ As is well known,^2–5^ in the propagation of CG chemical systems, one gains the possibility of sampling the configuration space more broadly, but one loses the effects from the fine-grained interactions that have been effectively removed. While the motion of the CG particles is also accelerated, this comes at the price that the dynamics is similarly skewed. In this paper, we reveal the degree to which observables are retained across the scales of two different now-standard CG approaches—namely MARTINI^3^ and DPD^4^— for a chosen complex system as a particular illustration for possible general application. Specifically, we develop and validate a DPD-4 parameterization for 1,2-dimyristoyl-*sn*-glycero-3-phosphocholine (DMPC) lipid vesicles—derived from MARTINI^3^ bead mappings and bonded terms (Sec. 2.3)—comparing its structural and dynamical properties against All-Atom and MARTINI models across a 12–60 nm size range.

Generally, energetics and other macroscopic variables in CG approaches are retained by construction, and so these models do preserve thermodynamic consistency. Here we aim to demonstrate the degree to which they retain structural and dynamical consistency. Structural consistency is defined as the agreement in local and global structural properties across the models, *e.g.*, when the structure function *g*(*r*) is in agreement between the two models at CG particle distances. Dynamical consistency is defined as the agreement in local and global time-dependent properties across the models, *e.g.*, when the correlation function *C*(*t*) is in agreement between the two models at time scales relevant to the motion of the CG particles. Note that the CG models are generally incapable of characterizing the spatio-temporal properties of the fine-grained particles as they have necessarily projected them out, and hence requiring such characterization is not part of the definition of structural or dynamical consistency.

The MARTINI force field was initially developed by Marrink *et al.*^3^ to simulate lipid bilayer systems and phase separation of chemical compounds. After improvement by them and many research groups, it has become one of the most widely used CG methods to simulate biomolecular systems, including lipid membranes, proteins, and hydrocarbons.^6–10^ The MARTINI force field is based on a four-to-one mapping—that is, every four heavy atoms are represented by a CG bead—where each CG bead is assigned to one of the four bead types—*viz.* polar (*P*), nonpolar (*N*), apolar (*C*), and charged (*Q*).^3,9^ The parameters are determined using a combination of both bottom-up and top-down approaches.^3,9^ The MARTINI simulation of lipid bilayer density profile has been reported to exhibit structural consistency between experiment and All-Atom simulations.^6^ Moreover, the lipid tail dynamics in MARTINI simulations^7^ was seen to be in qualitative agreement. By tuning the MARTINI parameters, the thermodynamics and phase behavior of ripple phase lipid membranes can qualitatively and quantitatively agree with All-Atom simulations.^10^ However, the existence of dynamical consistency in biomolecular systems arising from the MARTINI force field and other CG methods remains to be fully validated by All-Atom models. This paper provides at least a partial answer to this question in the context of a particular set of systems, thereby demonstrating “existence” of the correspondence though not necessarily its “necessity”.

Similar concerns arise for DPD. It is a CG method, based on underlying theory, developed to predict the hydrodynamic behavior of complex fluids and soft matter at long time- and length-scales with thermodynamic consistency.^11–13^ Similar to the MARTINI force field,^3^ the DPD CG scheme involves the grouping of neighboring atoms with similar chemical properties into larger beads of equal volume, reducing the particle counts accordingly.^5^ DPD uses a soft, linearly-repulsive conservative force, *F⃑*_*ij*_^*C*^, for pairwise nonbonded interactions between any bead *i* and neighboring bead *j* within a cutoff distance *r*_c_, allowing for larger timesteps and for simulations to reach time-scales inaccessible to All-Atom molecular dynamics (MD), and longer time-scales compared to MARTINI CG simulations (when DPD is implemented with higher number density beads):1
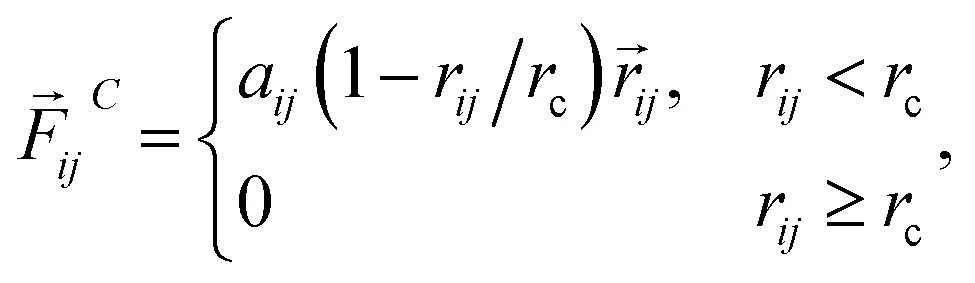
where *a*_*ij*_ is a parameter that defines the degree of repulsion between bead types and is optimized to reproduce the separation of hydrophilic and hydrophobic phases in bulk soft matter. Moreover, DPD is flexible in the number of heavy atoms grouped and the volume per bead; it can be readily re-parameterized accordingly.^14–18^ DPD simulations have been seen to capture the structural properties of lipid membranes,^4,17,19^ the swelling behavior of hydrogels,^20^ and the rheology of red blood cells through a judicious combination of forces:^18,21^ random and dissipative forces representing the fine-grained variables that are removed through coarse-graining,^12,13^ and conservative contributions representing the forces from nonbonded interactions, bond stretches, and angle bends between neighboring beads.

Both MARTINI and DPD CG simulations have been used in tandem to determine design rules for the surface chemistry of nanoparticles to control nanoparticle assembly and penetration into lipid membranes.^22–32^ The MARTINI force-field, for example, has been used to model lipid bilayer membranes, where the ripple phase of the membrane is not observed comparing to All-Atom simulations.^10^ The more recent MARTINI2 and MARTINI3 variants have also been vetted against All-Atom simulations of ternary lipid mixtures.^33^ On the other hand, DPD simulations can effectively capture monolayer lipid-water interfacial properties comparing to All-Atom simulations.^34^

The selected systems for the present illustration are vesicles made up of 270 to 2300 lipids and membrane-wrapped nanoparticles.^35^ Such systems are not arbitrary as they have argued to be potential vectors for biologically noninvasive transport and delivery of drug molecules and nanoparticles,^36–39^ motivated, in part, by promising biomedical applications such as molecular imaging and sensing, drug delivery, and photothermal cancer therapy.^40–42^ Systems of such lipid counts, correspond to vesicles in the size range of USUVs, which are 10 to 50 nm in diameter and known to be metastable and short-lived in biological environments.^43,44^ For example, they have been seen to be representative of physiologically important structures, including endocytic vesicles, exosomes, and viral particles.^45^ Recently, inclusion of anisotropy in the CG models has also been seen to enhance the accuracy of simulations of nanoparticle and vesicle interactions with lipid membranes.^46^ All of these simulations are inherently mesoscale: they predict molecular mechanisms of nanomaterial function and the empirical processes of lipid bilayers more generally, such as vesicle formation and fusion to membranes.^6,7,47^ They are often difficult to validate bottom-up from the atom-scale resolution using MD simulations^48^ or top-down by experiment.^49^

In the present work, we develop and validate a specific DPD-4 parameterization for DMPC lipid vesicles, demonstrating the structural consistency with the All-Atom model. We also demonstrate structural and dynamical consistency between MARTINI + P_4_ and DPD-4 models for this same system. Specifically, we report the structure and relaxation of lipid vesicles of varying sizes. We compare the structure and computational performance between All-Atom, MARTINI and DPD models for a 12 nm diameter USUV. The motivation for choosing this class of systems is that DPD is increasingly being used to model and describe local and global interactions at spatiotemporal scales much larger than what is accessible using All-Atom or MARTINI models. We also compare the structural and dynamical properties of MARTINI and DPD models for a 60 nm diameter vesicle which falls in the size range of SUVs. The relaxation of the DPD model is also compared to the trends surmised earlier from experiment.^50^

## 2 Methods

Here, we employ a suite of All-Atom MD, MARTINI, and DPD simulations to develop a mesoscale DPD lipid model. The latter is simultaneously fine-grained enough to capture local molecular-level structural changes and coarse-grained enough to access longer length-scales. Implementation of the DPD lipid model in USUVs of various small sizes has been validated by All-Atom and MARTINI simulations. It also reproduces the dynamics of larger SUVs (50–100 nm) reported in experiment.^50^

### 2.1 All-atom model and integrator

The All-Atom simulations^51^ are propagated using the CHARMM36 force field^52^ in LAMMPS.^53^ This choice of force field is appropriate because it has been extensively validated for phospholipid bilayers and vesicles; see *e.g.*, ref. 6, 10 and 52. Common All-Atom vesicle construction approaches, such as wrapping a planar bilayer onto a spherical mesh or lipid self-assembly, frequently lead to structural instabilities including membrane holes.^54^ To avoid this error, we instead employ a pre-packing method, implemented as a custom MATLAB/LAMMPS routine, to generate the initial vesicle structure: based on eqn (2), the total vesicle volume and per-lipid volume are used to determine the number of lipids required for a well-packed bilayer, ensuring that all lipids are placed with appropriate initial density prior to any MD equilibration. All simulations are performed at constant *NPT* at 300 K and 1 atm with a 2 fs time step. The All-Atom DMPC lipid vesicle is solvated in a cubic box with a minimum 15 Å TIP3P water cushion (between the vesicle and the sides of the periodic box) and 0.1 M KCl concentration. The number of TIP3P molecules inside the vesicle is calculated based on the density of water and the volume within the inner layer; see Fig. 1. The choice of USUV diameter (12 nm) is representative of observations because it is comparable to the average size (15 nm) reported in recent cryo-TEM image experiments.^55^ A 12 Å pairwise-interaction cutoff distance is used with a switching function that smoothly converged to zero between 10 to 12 Å. Electrostatic interactions are calculated using the particle–particle particle-mesh (PPPM) method. The SHAKE algorithm constrains hydrogen atoms to the heavy atoms. Three simulations are each run for 10 ns, followed by 5 ns production runs with data collected every 5 ps. We further confirmed the convergence to equilibrium within this time scale by propagating the 12 nm all-atom vesicle simulation by an additional 10 ns of production sampling. As reported in Fig. S6 and S7 in the SI), he resulting density profile, radial distribution function, and lipid tail end-to-end distance distributions are essentially unchanged.

**Fig. 1:**
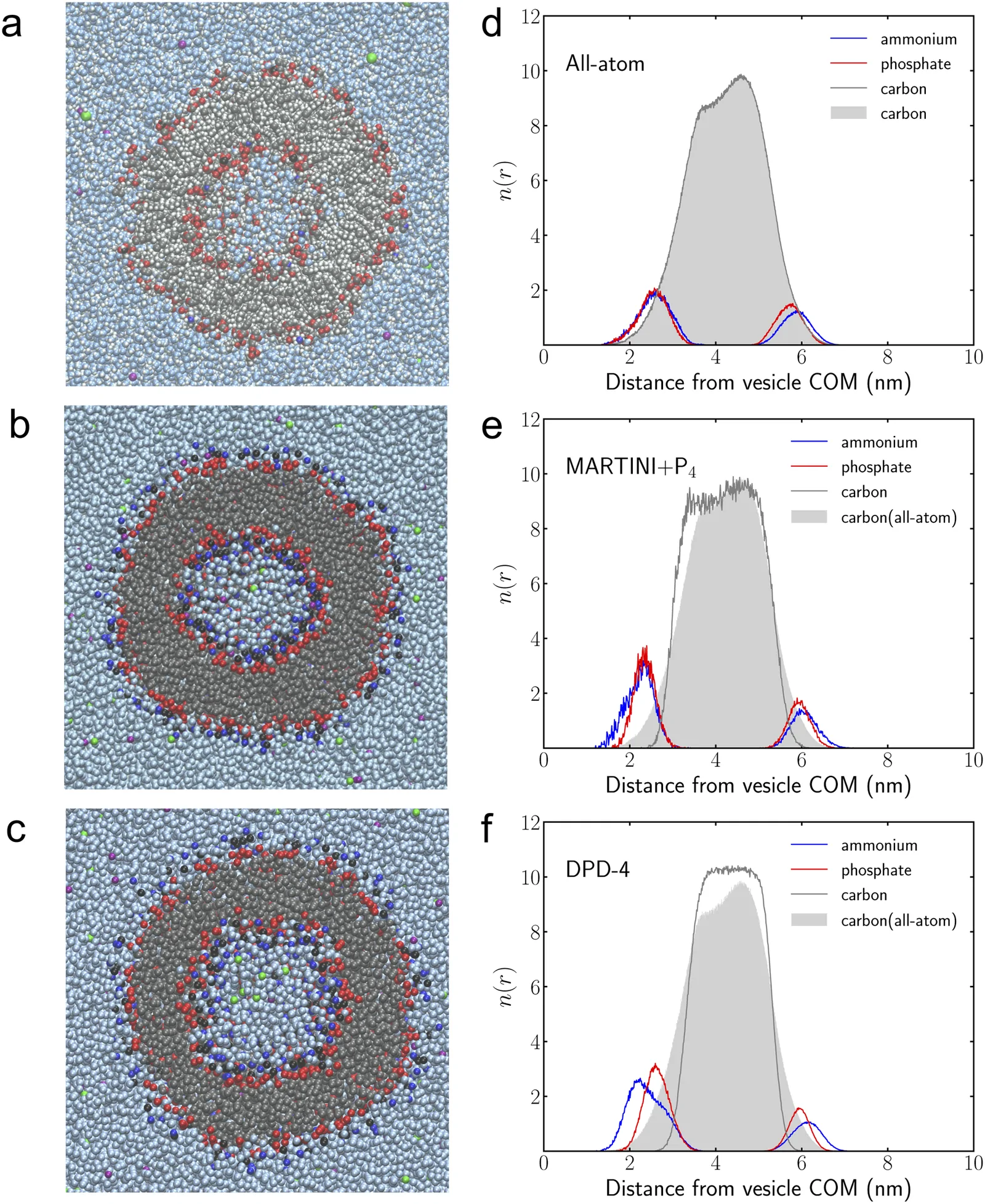
Cross section and radial distribution function between lipid functional groups and the vesicle COM for a ∼12 nm vesicle: (a) 640-DMPC lipid pre-packed using All-Atom MD, (b) 620-DMPC lipid MARTINI + P_4_, and (c) 620-DMPC lipid DPD-4. As all three vesicles shown here have a diameter of 12 nm, the length scale can be inferred from the vesicle diameter in all three images. The corresponding radial distribution functions are given on the right side of each scheme—*viz.* (d) All-Atom, (e) MARTINI + P_4_, and (f) DPD-4. The All-Atom vesicle was equilibrated in TIP3P solvent with K^+^ (purple) and Cl^−^ (green) counterions; carbon atoms are shown in gray, nitrogen in blue, oxygen in red, phosphorus in black, and hydrogen in white. In the MARTINI + P_4_ system, hydrophobic tail groups are in grey, ammonium head groups in blue, phosphate head groups in black. In the DPD-4 system, hydrophobic tail groups are in grey, ammonium head groups in blue, phosphate head groups in black.

### 2.2 MARTINI model and integrator

CG MARTINI simulations are carried out for four differently-sized DMPC lipid vesicles of diameters 12 nm, 17 nm, 25 nm, and 60 nm. In the simulations reported here, the differently-sized vesicles are modeled in solvents using forces based on the MARTINI 2.2 water model (P_4_) from CHARMM-GUI.^9,56^ The MARTINI 2.2 force field is chosen because it has been extensively validated for lipid bilayers and vesicles.^3,6,7^ In the SI, additional simulations are reported based on two alternative CG water models—*viz.* polarizable water (POL)^57^ and big multipole water (BMW)^8,58^—for comparison. In what follows we will refer to these three models as MARTINI + P_4_, POL-MARTINI and BMW-MARTINI, respectively.

The initial structures of the DMPC vesicles are generated using CHARMM-GUI^56^ with pores of sizes 5 to 10 Å, letting the water equilibrate inside and outside of the vesicles. Using this protocol, the MARTINI model could potentially relax to a different local equilibrium than that found by the AA procedure, and thereby affect comparisons between them. To mitigate this possibility, both systems were allowed to relax for relatively long time before collecting statistics so as to reach their global equilibrium albeit with respect to their particular force field. The vesicle is solvated with a water layer of thickness 40 Å on all sides and an ionic(NaCl) strength of 0.1 M. A 20 fs integration time step is used with the Verlet cutoff scheme (neighbor list updated every 20 steps). A 11 Å cutoff for both van der Waals (with a potential-shift-Verlet modifier) and Coulomb interactions. The latter is treated using the reaction-field method with a relative dielectric constant of *ε*_r_ = 15. The no-bond-constraint algorithm is applied. The temperature is maintained at *T* = 303.15 K using a velocity-rescaling (v-rescale) thermostat with a time constant τ_*T*_ = 1.0 ps, and the pressure is maintained at *P* = 1 bar using a Parrinello–Rahman barostat (*τ*_P_ = 12.0 ps). For 12, 17, and 25 nm vesicles, isotropic pressure coupling is applied through a compressibility of 4.5 × 10^−5^ bar^−1^. For 60 nm vesicles, semi-isotropic pressure coupling is applied through a compressibility of 3 × 10^−4^ bar^−1^. Once the vesicle is preassembled, the equilibration is relatively fast, requiring a total simulation time of not much more than 1000 ns. The final 40 ns of the trajectory is used for data analysis.

### 2.3 DPD model and integrator

DPD is a mesoscale simulation methodology and not just the underlying fixed force field. In this work, we employ two specific parameterizations for the DMPC vesicles: In DPD-4 and DPD-3 approximately four^5^ and three^30^ heavy atoms, respectively, are mapped onto a single bead.

Our parameters for the lipid-bilayer systems of interest in this work were modified from a DPD-4 lipid model developed recently using existing MARTINI model potentials as a ref. 5 This approach was inspired by Gao's model^59^ for DMPC lipids. We included additional bonded interaction parameters for the angle along the lipid head groups, which are consistent with the MARTINI representation,^59^ but not previously defined. This allowed us to accurately represent the rigidity of the lipids and incorporate the finer-grained, molecular-level structural properties of the fully saturated DMPC lipids into the DPD-4 lipid model. We also included electrostatic interactions,^60^ where the detailed CG mapping schemes for both DPD-4 and DPD-3 models are shown in Fig. S1 of the SI. The force field parameters for the DPD-4 and DPD-3 modes are available in Tables S1 and S2, S6 and S7, respectively, in the SI. The pairwise nonbonded cutoff distance is *r*_c_ = 1.0 in DPD reduced units for all bead pairs, corresponding to *r*_c_ = 7.11 Å in physical units for the DPD-4 mapping used throughout (Sec. 3.3). Because the DPD-4 bonded and nonbonded parameters are derived directly from MARTINI potentials, structural agreement between the two models at the bead level (Sec. 3.3) is not a fully independent validation, and should be expected unless the different forms of the interactions lead to deviations.

For our DPD-4 simulations, four different sized vesicles of diameters 12 nm, 17 nm, 25 nm, and 60 nm were simulated using LAMMPS.^53^ The vesicles were surrounded by the majority of 4 water molecules beads and no counterions. For the 12, 17, and 25 nm vesicles, initial DPD bead positions were generated with a custom Python script that maps the corresponding CHARMM-GUI-generated MARTINI structure (Sec. 2.2) onto the DPD-4 bead representation. The 60 nm vesicle was instead packed independently using Packmol,^61^ as described in Sec. 3.3. For the 12, 17, and 25 nm vesicles, trajectories were generated with an integration time step of 0.003 DPD time units. Each simulation was equilibrated for at least 1 000 000 DPD time units in total, followed by a 500 000 DPD time unit production run with data collected every 1000 integration time steps—*viz.* every 3 DPD time units—for analysis. For the 60 nm vesicle, a smaller integration time step of 0.001 DPD time units was used instead, chosen so that saved configurations correspond to round real-time intervals (*e.g.*, 1 ns, 10 ps); the corresponding equilibration, production, and save-interval lengths for the 60 nm system are given in Sec. 3.3. For each vesicle size and model, multiple independent trajectories were generated. As the analytics were consistent across replicas, only the values from a representative trajectory are reported here for each case. The uncertainties (Sec. 3.3, SI) reflect block-averaging statistics within the selected trajectory. Based on previous DPD mapping approaches,^5^ we estimate that 1 DPD time unit corresponds to 91.4 ps in real time. This is significantly longer than typical All-Atom MD simulations of similar systems, which are often confined to the nanosecond timescale due to computational resource limitations.

## 3 Results and discussion

### 3.1 Lipid counts in inner and outer layers

We compare four different sized USUVs with diameters of 12 nm, 17 nm, 25 nm, and 60 nm. The distribution of lipids between the inner and outer numbers was determined using theoretical predictions, validated against CHARMM-GUI generated models. As derived in the SI, the ratio between the number of lipids in the outer and inner layers is2
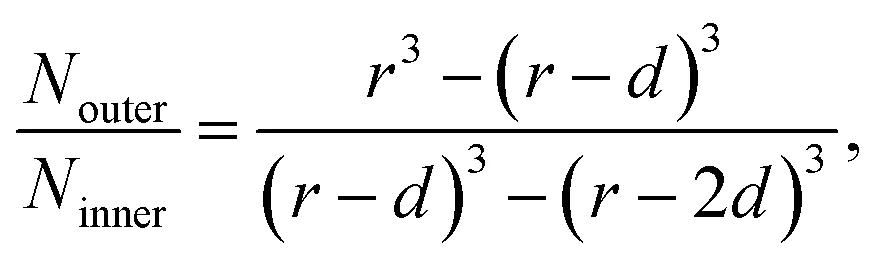
where *r* represents the vesicle's outer radius, and *d* = 2 nm is half of the average width of the bilayer. As shown in Fig. 2, this relationship exhibits non-linear behavior, with the ratio's rate of change decreasing as the vesicle radius increases. The black solid curve represents the theoretical prediction of eqn (2), while the red dashed line corresponds to the simulations integrated over the DPD-4 and MARTINI + P_4_ force fields. The striking agreement between these curves validates the physical accuracy and robustness of our model in reproducing the total lipid counts across both layers with only a small deviation when *N*_inner_ is small and hence the diameter is small. The latter is confirmed in Fig. S2 in SI where the ratio *N*_outer_/*N*_inner_ for USUVs is seen clearly to match eqn (2) better than the exponential fit at <20 nm.^35^ In the 12 nm vesicle, the lipid counts in the inner and outer layers are shown in Table S8. Furthermore, the lipids in the All-Atom simulations also cover the same area, 0.64 nm^2^, per lipid with both DPD-4 and MARTINI + P_4_ simulations. These values for the ratio of outer to inner lipids and area per lipids were used to construct and equilibrate pre-packed All-Atom 640-DMPC lipid vesicles.^61^

**Fig. 2:**
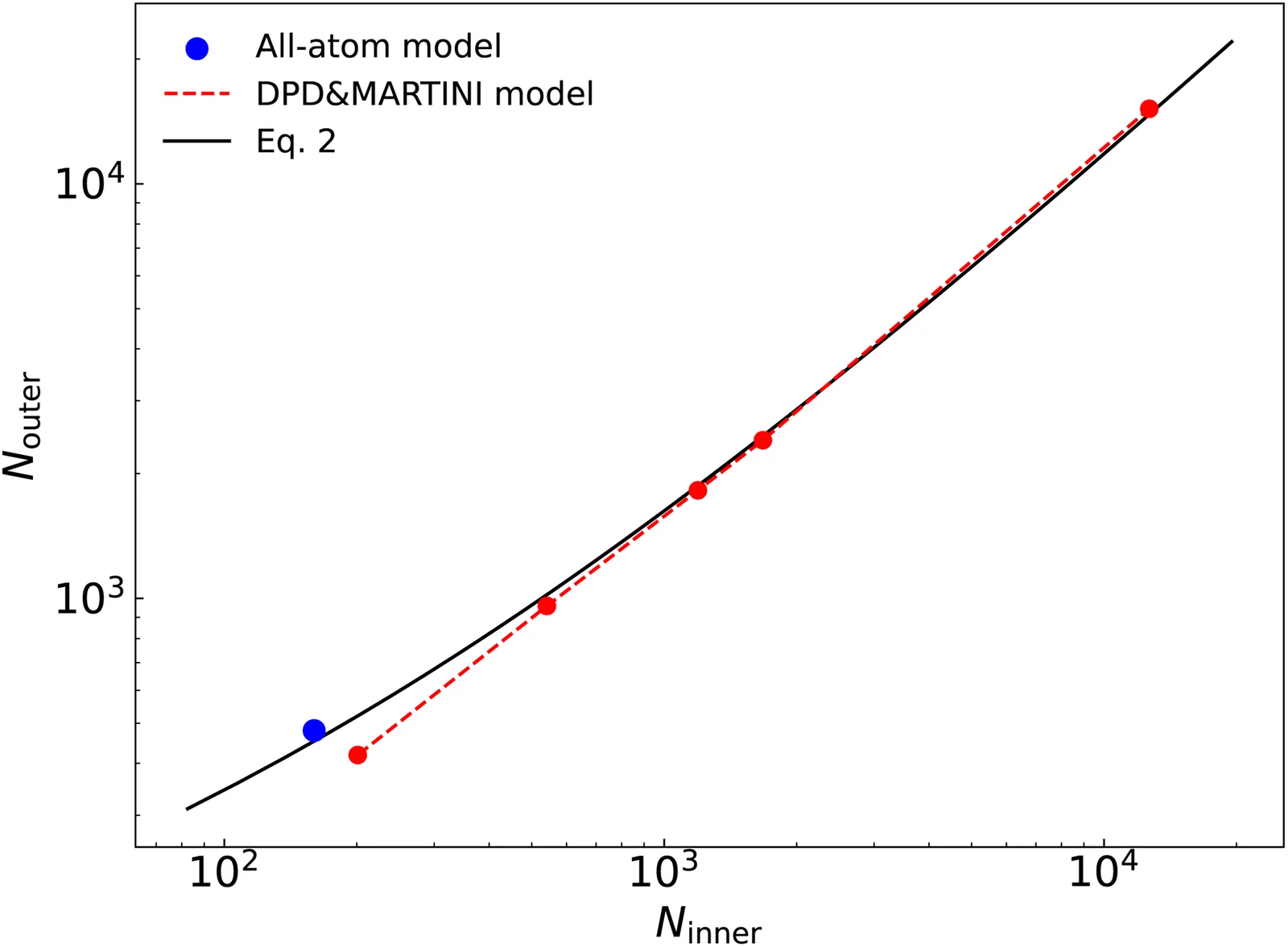
Number of lipids in the outer (*N*_outer_) *versus* inner (*N*_inner_) layers of USUVs. The vesicle membranes containing total lipid counts of 620, 1500, 3016, 4084 and 27 848 correspond to vesicle diameters of 12, 17, 22, 25 and 60 nm respectively. The DPD-4 and MARTINI + P_4_ CG models (in red) resulted in the same numbers, and are compared to the All-Atom 12 nm model (in blue). The theoretically expected nonlinear result according to eqn (2) is shown in black.

### 3.2 Computational performance

The performance of a 12 nm vesicle system is evaluated using three simulation approaches: All-Atom, MARTINI + P_4_, and DPD-4; see Table 1. For All-Atom simulations, a 5 ns trajectory required approximately 127 hours to complete on 144 cores across 3 nodes, utilizing Intel(R) Xeon(R) Gold 6248R CPUs at 3.00 GHz, achieving a performance of 0.918 ns day^−1^. Extrapolating the All-Atom cost rate measured for the 12 nm vesicle, for the 60 nm system size (27 848 *vs.* 640 lipids, a 43.5-fold increase in particle number), and assuming this cost scales approximately linearly with particle number, gives a projected cost of ≈159 000 core-hours ns^−1^. This extrapolation is necessarily approximate, since All-Atom cost, particularly for long-range electrostatics, does not scale strictly linearly with system size; however, any deviation from linear scaling would increase rather than decrease the projected All-Atom cost, so the qualitative conclusion—that All-Atom simulation of the 60 nm vesicle is computationally intractable at present—supports why such simulations are not reported below.

**Table 1:** Computational performance comparison of All-Atom, MARTINI, and DPD simulations for the 12 nm DMPC vesicle

	All-atom	MARTINI + P_4_	DPD-4
Software	LAMMPS 21Jul2020	GROMACS 2021.4	LAMMPS 21Jul2020
Hardware	Intel Xeon Gold 6248R	Intel Xeon Gold 6248R	Intel Xeon Gold 6248R
Number of cores (nodes) used	144 cores (3 nodes)	48 cores (1 node)	48 cores (1 node)
Wall-clock time (h)	127	7.24	1.08
Total simulation time (ns)	5	1000	137
Speed (ns day^−1^)	0.918	3314	3028

In contrast, the MARTINI model, simulated using GROMACS 2021.4 on 48 cores across a single node with the same CPUs, completed 1000 ns in approximately 7.24 hours, corresponding to a performance of 3314 ns day^−1^. For the DPD-4 model, where 1 DPD time unit corresponds to 91.4 ps according to Wang and Hernandez,^5^ and hence each timestep of 0.003 DPD time units equates to 0.273 ps. Simulations of this model were performed on 48 cores across a single node and completed in 1.08 hours, yielding an impressive performance of 3028 ns day^−1^. For example, for the 60 nm production run using DPD-4 only ≈59 core-hours ns^−1^ were required. This is roughly three orders of magnitude (∼2, 700-fold) better than the performance extrapolated for the All-Atom model above, and is likely even better than this because the latter assumed unachievable linear scaling. Details of the relative performance are summarized in Table 1, but a specific numerical ratio is not reported because the All-Atom model required more cores and nodes than were used for the CG models while reaching times less than 1000 times smaller in a day of compute. Thus, the DPD and MARTINI CG approaches, exhibit a significant computational advantage relative to the All-Atom model. It is notable that the performance of the MARTINI CG model may be even better than it appears, as its effective simulation time is typically accelerated by a factor of ∼4 due to the coarse-graining of fine-grained motion. The DPD model time scale is parameterized to match atomistic diffusion directly, and thus does not require additional time rescaling. The performance reported here for DPD-4 simulations is similar, though a little slower, to what we reported for the DPD-4 simulations of the smallest vesicle and is also effectively slower than MARTINI, but DPD-*N* outperforms MARTINI for larger vesicles because the DPD-*N* CG time step and bead size can be set to be larger for a higher degree of coarse-graining of *N*: 1 when *N* ≫ 4.^5^ Based on these performance trends, we recommend MARTINI when finer local structural detail and established force-field validation are needed at moderate system sizes and sub-microsecond timescales, and DPD (with *N* ≫ 4 for a coarser mapping) for larger systems, where its bead size and timestep can be scaled up further to give a growing performance advantage over MARTINI.^5^

### 3.3 Vesicle structure

We now compare the All-Atom, MARTINI + P_4_ and DPD-4 model vesicles to establish the degree with which they are in structural consistency.

The All-Atom 640-DMPC lipid vesicles, pre-packed initially with lipid tails fully extended, equilibrated to the same size of approximately 12 nm, along with similar density profiles of major lipid functional groups and hydrophobic bilayer thickness of 4 nm in agreement with experiments^62^ (Fig. 1 and S12 in the SI). A possible caution in interpreting the All-Atom results lies in the inherent challenges to equilibration of All-Atom vesicle systems. The lipid redistribution and water exchange across the bilayer leaflets are of particular concern as they, occur on timescales beyond those typically accessible in atomistic simulations.^54^ In the present work, local structural stability of the All-Atom vesicle over the simulated window was confirmed by convergence of the total energy, the absence of any observed lipid flip-flop events (*i.e.*, no change in the initial leaflet lipid counts over the simulated window), absence of membrane defects upon visual inspection (VMD), and radial distribution functions showing well-defined inner and outer leaflet positions of lipid headgroups and tail carbons consistent with an intact bilayer structure. The reported 10 ns equilibration and 5 ns production window followed additional staged relaxation, carried out as a sequence of restarted NPT/Langevin runs, during which convergence of the total energy was monitored and confirmed prior to the final reported window. Data were collected over the final 5 ns of the production run. While these metrics confirm local structural stability, slower processes such as transbilayer lipid flip-flop may remain incompletely sampled regardless of the total equilibration time invested. Such processes occur on time scales orders of magnitude beyond what is accessible even to a substantially extended conventional All-Atom trajectory. Our All-Atom benchmarks should therefore be interpreted as an intermediate-time, locally-relaxed structural reference only—suitable for the density profiles, radial distribution functions, and *r*_ee_ distributions reported here—and not as a benchmark for global vesicle equilibration, leaflet composition, vesicle shape, curvature stress, hydration, or slow dynamical relaxation. The dynamical comparisons in Sec. 3.5 instead use literature All-Atom planar-membrane data,^63^ which is not subject to this same short-time equilibration limitation.

The site–site radial distribution functions *g*(*r*) between phosphorus head group sites (P–P) further confirm structural consistency across models (S4): all three models reproduce the primary and secondary coordination peaks, with the sharper peaks in MARTINI reflecting reduced coarse-grained degrees of freedom and the broader features in DPD consistent with its soft interaction potential. Beyond visual comparison, a Kolmogorov–Smirnov (KS) test between the All-Atom and each of the MARTINI + P_4_ and DPD-4 density profiles for each functional group (Table S3 in the SI) quantifies this structural consistency: DPD-4 is closer to the All-Atom reference for the ammonium and phosphate head groups (*D* = 0.16–0.20), while MARTINI + P_4_ is closer to the All-Atom reference for the carbon tail region (*D* = 0.04 *vs. D* = 0.10 for DPD-4). The head-group density profiles are bimodal, with well-separated inner- and outer-leaflet peaks whose positions agree with the All-Atom reference to within 0.5 nm (inner leaflet) and 0.2–0.3 nm (outer leaflet) for both MARTINI + P_4_ and DPD-4. The corresponding inner-to-outer peak separation is 3.1–3.4 nm for the All-Atom reference and 3.3–3.9 nm for the two CG models. Statistical uncertainty for these 12 nm density profiles, obtained by block-averaging within the selected trajectory for each model, is reported as a shaded band in Fig. S5, and the corresponding block-to-block distribution of the layer separation and hydrophobic-core width is reported in Table S4 (both in the SI). As with the *r*_ee_ distributions presented below, the larger DPD-4 layer separation and its larger *D*-values in the KS statistic for the head-group profiles likely reflect the same soft, purely repulsive pairwise potential and coarser 4 : 1 mapping, which smear out sub-nanometer structure relative to the sharper, explicit-angle-term MARTINI potential. Retuning the head-group *a*_*ij*_ values is a plausible route to reducing this deviation, but we leave this for future work. The aspherical shape is due to the high bending resistance of smaller vesicles with high surface curvatures.^64,65^ The All-Atom, MARTINI + P_4_ and DPD-4 models are consistent not only in the overall radial density profile (Fig. 1), but also in the local structure within vesicles, as measured by the effective lipid tail end-to-end distance, *r*_ee_ (Fig. 3). Note that for consistency in the comparison across the models, *r*_ee_ is calculated by taking the difference between the radial coordinates relative to the vesicle COM of the lipid glycerol bead and the final lipid tail bead in the CG models, and the COMs of the atoms grouped within the corresponding CG beads for the All-Atom system. It is also notable that the MARTINI + P_4_ model matches the All-Atom structure for the 12 nm vesicle in Fig. 1 particularly well.

**Fig. 3:**
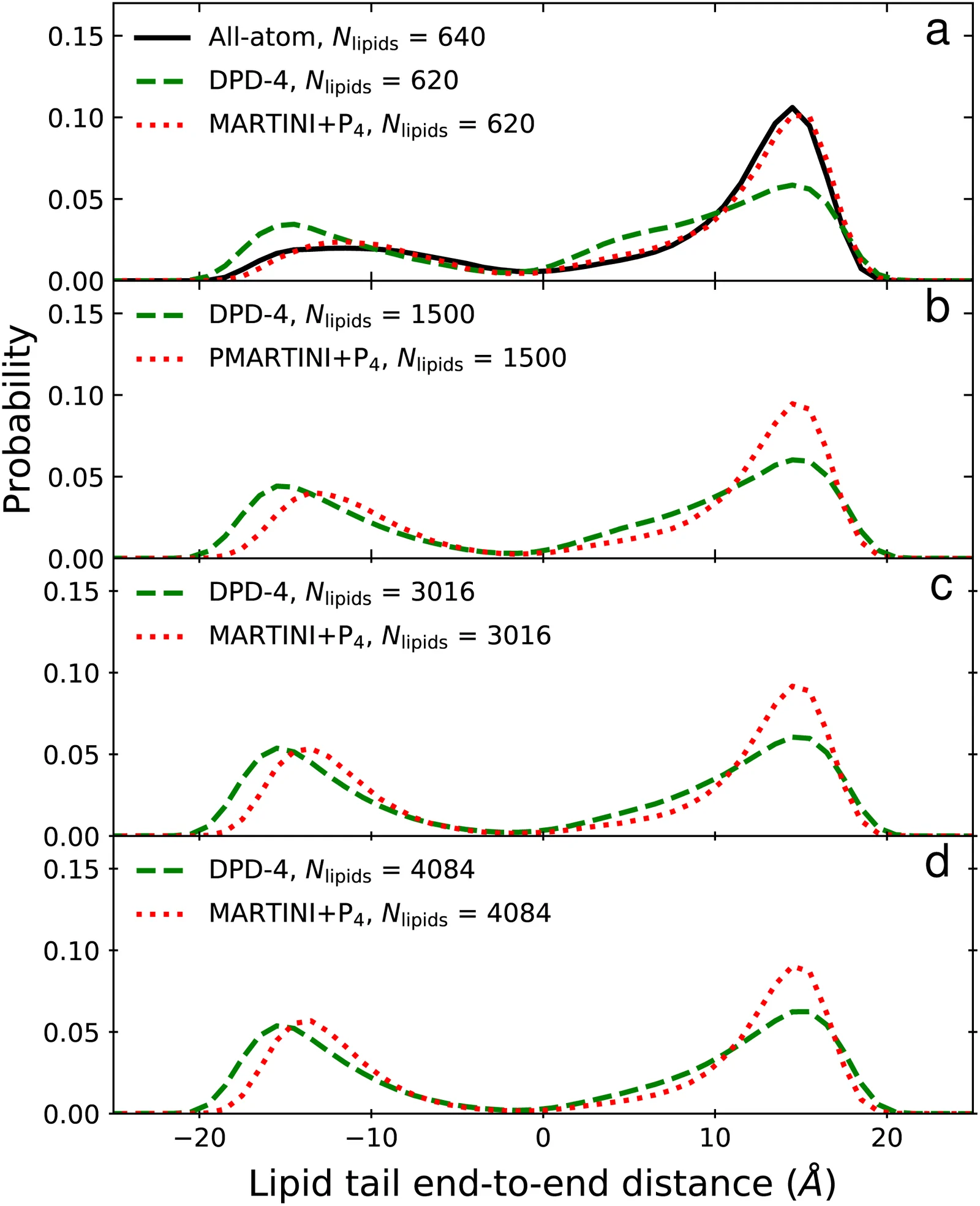
Probability distributions of lipid tail end-to-end distances, *r*_ee_, in the inner (negative values) and outer (positive values) layers of 12 nm, 17 nm, 22 nm and 25 nm diameter vesicles in panels (a), (b), (c) and (d), respectively, The DPD-4 and MARTINI + P_4_ distributions are shown in green dashed and red dotted curves. The *r*_ee_ distribution in All-Atom DMPC-lipid vesicles obtained only for a 12 nm vesicle is shown in solid black in panel a. The probability distributions are normalized by the total number of frames sampled such that a frequency of 1.0 indicates one instance in every frame. The number of lipids in the vesicles for each case is noted in the legend, but the distributions are normalized relative to these totals.

The end-to-end distance, *r*_ee_, is a measure of the radial positional difference between the glycerol backbone and the lipid tails in a lipid bilayer. Specifically, it quantifies the degree of tilt in the lipid tails relative to the glycerol bead, and thus serves as a descriptor of lipid tail orientation. It is defined as the difference in radial distances of the glycerol bead (bead-level COM of 3,Na and 4,N_a_ in the top scheme in S1) and tail beads (bead-level COM of 8,c and 12,c in the top scheme in S1) from the vesicle's overall COM, distinguished below as **r**_ves_ from the bead-level COMs above. That is,

3 *r*_ee_ = *r*_glycerol_ − *r*_tail_

where *r*_glycerol_ and *r*_tail_ are the Euclidean distances of the glycerol bead and the tail bead from the vesicle's COM, respectively:

4 *r*_glycerol_ = ‖**r**_glycerol_ − **r**_ves_‖

5 *r*_tail_ = ‖**r**_tail_ − **r**_ves_‖

A positive *r*_ee_ indicates that the glycerol bead is positioned further from the vesicle COM than the tail bead (*r*_glycerol_ > *r*_tail_), which corresponds to lipids in the outer leaflet of the bilayer. Conversely, a negative *r*_ee_ suggests that the glycerol bead is closer to the COM compared to the tail bead (*r*_glycerol_ < *r*_tail_), which corresponds to lipids in the inner leaflet.

The absolute magnitude of |*r*_ee_| provides further insight into the degree of lipid tail extension and tilt. When |*r*_ee_| is large, the difference between *r*_glycerol_ and *r*_tail_ is more pronounced, indicating that the tail is relatively extended and exhibits a smaller tilt angle. In contrast, a small |*r*_ee_| suggests that the tail bead is positioned closer to the glycerol backbone in the radial direction, leading to a more tilted tail conformation. When *r*_ee_ ≈ 0, the glycerol and tail beads are nearly equidistant from the COM, representing a highly tilted or folded lipid structure.

The All-Atom, MARTINI + P_4_ and DPD-4 systems generally have similar population distributions of lipid configurations in *r*_ee_. At lower *r*_ee_, the MARTINI model appears to be in slightly better agreement than the DPD-4 model with the All-Atom model, perhaps because it includes some of the shorter length scale behavior. Low *r*_ee_ values suggest lipid tails with greater tilt or splayed configurations to induce curvature within the smallest vesicles of the USUV range (Fig. 3). Meanwhile, the DPD-4 model captures the structural changes within vesicles with increasing vesicle size and decreasing curvature. In the larger 17 and 25 nm vesicles, the population of lipids with lower magnitude *r*_ee_ significantly decreases, and the population of lipids with high *r*_ee_ (*i.e.* individual lipids with tightly packed tails) is sharpest for 25 nm vesicles as the outer surface becomes increasingly flat (Fig. 3). Nevertheless, we acknowledge that the *r*_ee_ distribution of DPD-4 in Fig. 3a overestimates the population at lower *r*_ee_. This discrepancy likely reflects DPD-4's softer, purely repulsive pairwise potential and coarser 4 : 1 mapping, which smear out the finer tail tilt and packing detail that MARTINI's explicit angle terms and sharper nonbonded potential retain. This effect is most pronounced in the more tightly curved inner leaflet, and also contributes to the residual differences between DPD-4 and MARTINI at the outer leaflet across the vesicle sizes studied. Retuning the tail-bead *a*_*ij*_ or bond–angle parameters is a plausible route to reducing this discrepancy, but we leave such parameterization for future work. Additional comparisons of *r*_ee_ are provided in SI Section S3.

### 3.4 Structural consistency

Our structurally benchmarked DPD lipid parameters can now be used to construct larger lipid-bilayer systems that are more accessible to experimental interrogation, and thereby establish their dynamical consistency with experiment. First, from the radial distribution functions between lipid functional groups and the vesicle COM across the range of USUVs modeled, we determined the size of the vesicle inner layers in Fig. 1. Dividing the size of the inner layer by the number of lipids in the inner layer, we found that the inner layer surface area to lipid ratio is consistent across sizes and in accord with experimentally determined values for the area per lipid, specifically 0.66 nm^2^/DMPC lipid.^16^ This empirical choice of surface area of the lipid bilayer, thus, dictates the size of the inner layer of the vesicle. Meanwhile, the dynamics of SUVs as small as 60 nm have been measured using neutron spin-echo (NSE) spectroscopy, as recently reported by Hoffmann *et al.*^50^ To construct a 60 nm vesicle in our simulations, we first subtracted 8 nm from this desired size to account for the two bilayers of 4 nm thickness across a vesicle's diameter. We then used the area per DMPC lipid to pack the corresponding number of lipids in the inner layer of the vesicle with 52 nm in diameter. Beyond the USUV range however, the relation between the number of lipids in the inner and outer layer of vesicles should not be linear as shown in Fig. 2, since the ratio should asymptotically approach unity with decreasing vesicle curvature towards planar membranes. Indeed, Stelter and Keyes^35^ earlier suggested a different nonlinear form for this ratio, namely,6
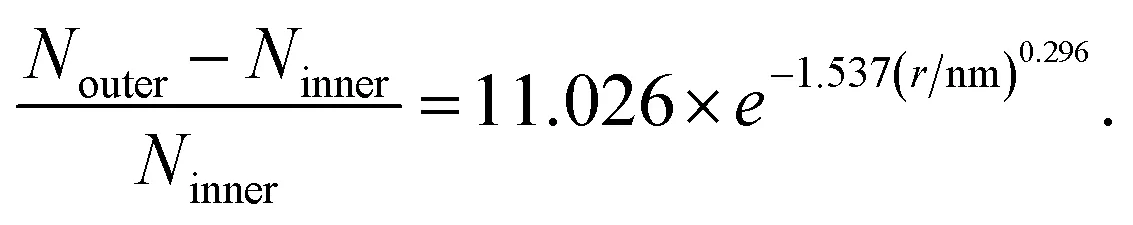
We show in Fig. S2 in the SI that eqn (6) and (2) agree for the 60 nm vesicle, and the former is in slightly worse agreement at USUV sizes. This consistency assures us that we can use these expressions to determine the numbers, *N*_inner_ and *N*_outer_, of lipids in the outer layers of the vesicle. In the present case, *r* is the inner layer radius of 25.8 nm, and the number of lipids in the inner layer *N*_inner_ = 12, 673 is determined from the area per lipid. The result is *N*_outer_ = 15, 175 with a total number of 27 848 lipids in the 60 nm vesicle. These vesicles were constructed using Packmol^61^ with the number of water beads inside the vesicle determined by a number density per volume *ρ* = 3 (*i.e.*, 3 beads per *r*_c_^3^ in reduced DPD units, following the standard convention of Groot and Warren,^13^ where *r*_c_ is set to 7.11 Å as established in ref. 5), as is typical^23^ for the resolution and parameterization employed in this work. Three trajectories were each run for 500 000 DPD time units followed by a 25 000 DPD time production run with data output every 100 DPD time units for analysis. Representative snapshots of the MARTINI + P_4_ and DPD-4 CG 60 nm vesicles from their respective trajectories are shown in Fig. 4.

**Fig. 4:**
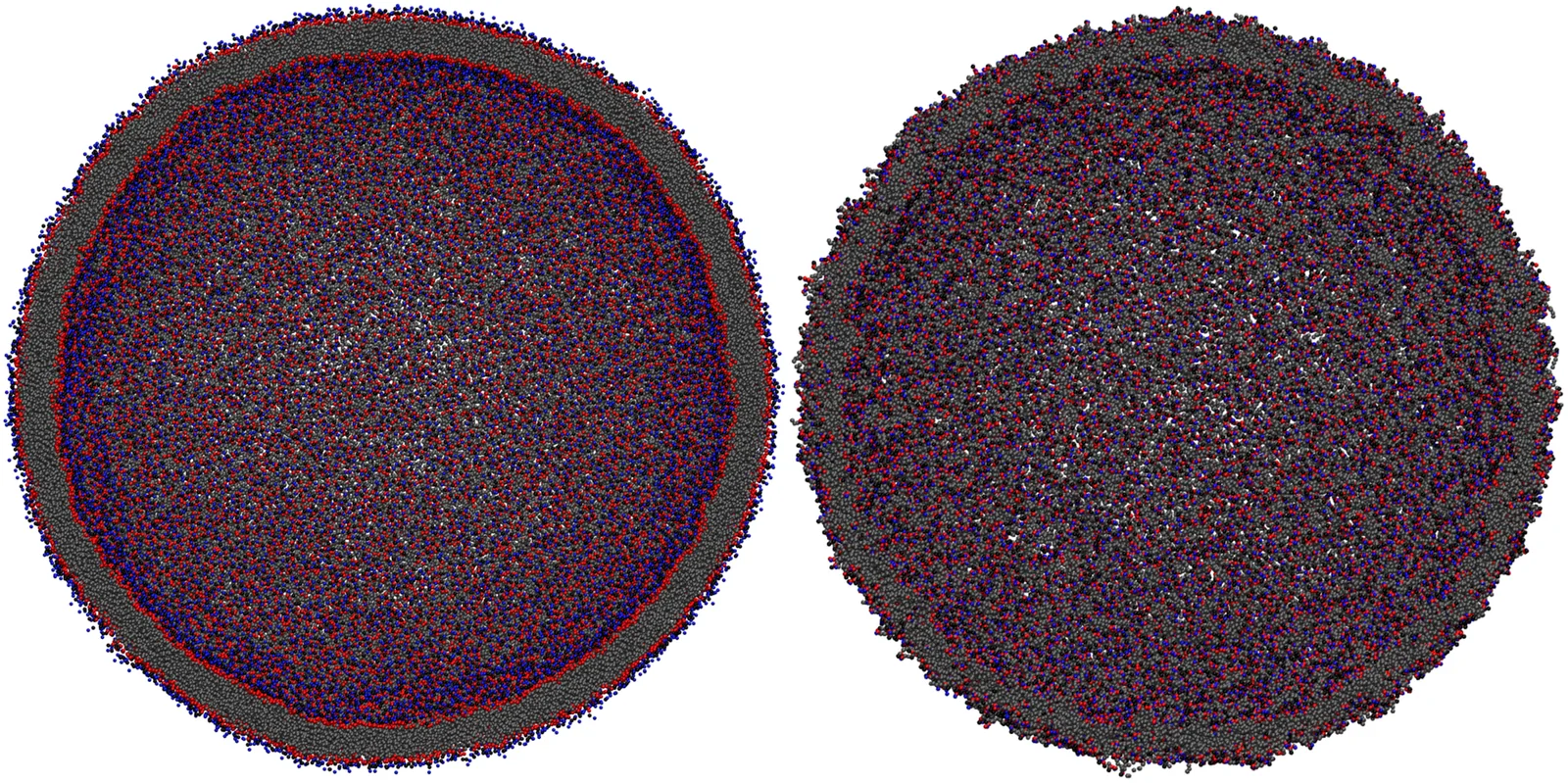
MARTINI + P_4_(Left) and DPD-4(Right) CG 27,848-lipid, 60 nm small unilamellar vesicles shown from the hemisphere to reveal the cross section along an equatorial plane. Ammonium head groups are in blue, phosphate head groups in black, glycerol groups in red, and hydrophobic tail groups in gray for MARTINI + P_4_. Ammonium head groups are in blue, phosphate head groups in orange, glycerol groups in red, and hydrophobic tail groups in gray for DPD-4 water beads are removed for clarity.

### 3.5 Dynamical consistency

Membrane undulations and their relaxation dynamics can be captured in the intermediate scattering function *F*_s_(*q*, *t*). The latter can be measured directly from NSE spectroscopy experiments or indirectly computed through the spatial Fourier transform of the self-component of the pair correlation function *G*_s_(*r*, *t*) from simulation. Zilman and Granek^66^ found that the relaxation dynamics of planar membranes follows a stretched exponential decay, *F*_s_(*q*,*t*) ∼ exp(−(*Γq*^3^*t*)^2/3^) across a range of wave numbers *q* at a rate of *Γ* ∼ 0.025(*k*_B_*T*)^3/2^/(*κ*^1/2^/*η*), where *k*_B_ is the Boltzmann constant, *κ* is the bending modulus and *η* is the solvent viscosity. In NSE spectroscopy on 60 nm SUVs, the relaxation dynamics of SUVs was also seen to fit the Zilman–Granek model. Thus SUVs thereby exhibit similar dynamics to planar membranes despite having greater curvature.^50^ While the SI reports the DPD friction coefficient, cutoff, dielectric constant, and water bead nonbonded parameter used in the simulations, we have not independently calibrated the solvent viscosity *η* or bilayer bending modulus *κ* in our DPD trajectories to the expected values. The prefactor *Γ* below is therefore reported as an empirical fit parameter rather than one derived from independently measured *η* and *κ via* the Zilman–Granek relation above. This is sufficient to establish the qualitative trends reported here.

To confirm whether the CG SUVs exhibit similar dynamics, we thus aim to validate that they follow the form of the Zilman–Granek model equation. We first compute the self-component of the van Hove function,7
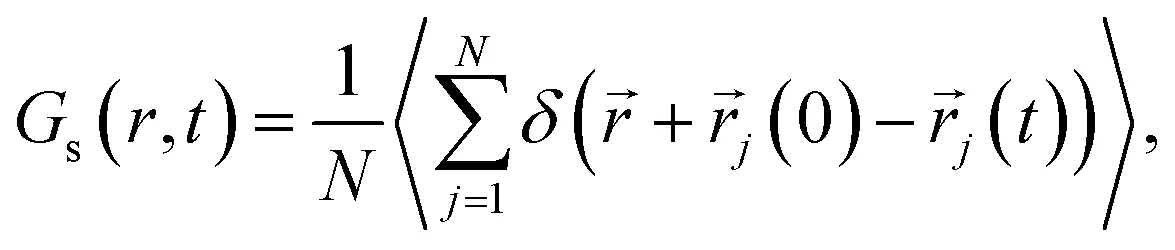
which is the probability of finding bead *j* a distance of *r* (≡|*r⃑*|) away from its initial position *r⃑*_*j*_(0) at time *t* caused by the displacement *r⃑* in any arbitrary direction, for each hydrophobic bead *j* in the tails of each lipid. Prior to using *r⃑*_*j*_(*t*), the vesicle's own COM translation is subtracted and its rigid-body rotation is removed by a per-frame alignment of the tail beads to the initial frame. Thus *G*_s_(*r*, *t*) reflects only the beads' motion relative to the vesicle itself, not its whole-vesicle drift or tumbling. To ensure correspondence in the comparison between All-Atom structures and the coarse-grained methods, and to avoid conflicts in representability, structures along the All-Atom trajectory are first mapped onto the analogous coarse-grained bead representations. Namely, atomic groups are grouped into beads associated with their COM positions. Consequently, the sum in eqn (7) runs over the same set of effective sites in all three models. Quantitative comparison between MARTINI + P_4_, DPD-4, and All-Atom models using the van Hove function are shown in Fig. 5. The MARTINI + P_4_ exhibits faster dynamics consistent with the well-known ∼4-fold acceleration of the MARTINI force field.^67^ At short times, MARTINI, even with the heuristic time rescaling, shows larger KS-test deviations than DPD-4 from the relative to the All-Atom reference (Table 2). Thus the former is at best approximate for curved-vesicle undulating dynamics. Nevertheless, the DPD-4 and rescaled MARTINI + P_4_ both show dynamical consistency with the All-Atom model, at least for the longer times. Note that any discrepancy in the comparison to the scattering function of the Zilman–Granek model is not a result of a single overall time scaling because such scaling is optimized in the fitting parameters of the model. More detailed comparison results for the van Hove function are shown in Fig. S8 in the SI. The lipids in the DPD-4 model of the 60 nm vesicle had a similar displacement probability profile as that of lipids across the span of 1 ns All-Atom membrane simulations.^63^ This time scaling was found by matching the DPD van Hove displacement profile to the All-Atom reference as shown in Fig. 5 and S8 in the SI. The fixed DPD unit conversion (1 DPD unit = 91.4 ps) found earlier in Sec. 2.3 for the smaller vesicles evidently does not extrapolate to the larger vesicles. Use of the DPD-3 model led to preservation of dynamical consistency across the AA and CG scales; see Fig. S10 to S16 in the SI. Specifically, the use of the KS test between the AA and CG scales reported in Table 2 reveals larger deviations resulting from MARTINI + P_4_ than from DPD-4, and the latter are generally within the criterion for statistical significance. One possible origin for the deviation found from the MARTINI model is the fact that they can sometimes fail to properly account for the decomposition between enthalpy and entropy as recently reported.^68,69^ In summary, the DPD-4 model shows somewhat better agreement with the All-Atom van Hove functions reported by Vattulainen and coworkers.^63^

**Fig. 5:**
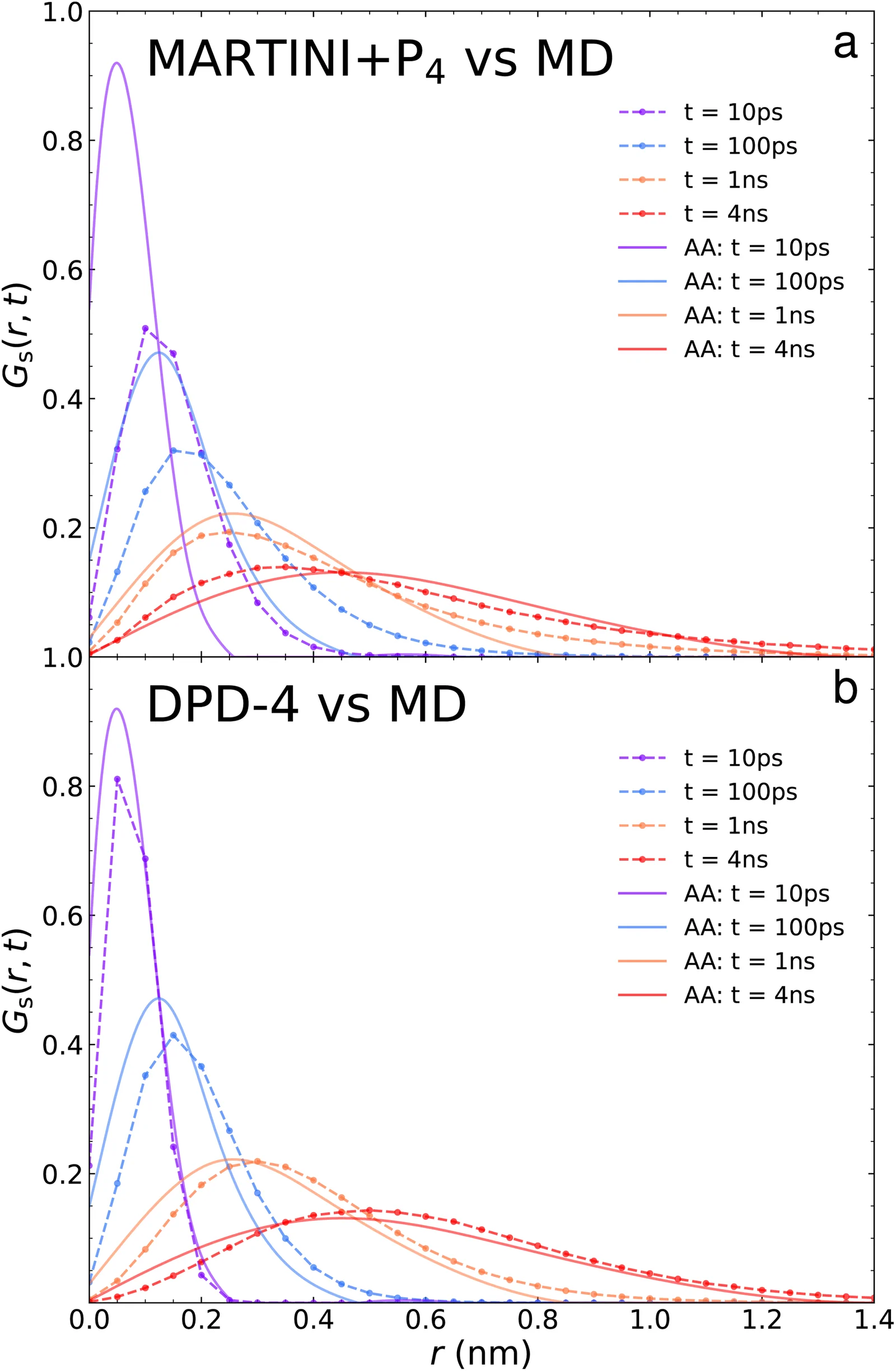
The van Hove functions *G*_s_(*r*, *t*) of eqn (7) for the lateral displacement *r* in time *t* of lipids relative to a surface obtained using MARTINI + P_4_ (panel (a)) and DPD-4 (panel (b)) for a 60 nm vesicle (dashed curves) are compared with those reported in ref. 63 for the All-Atom (AA) MD simulations of lipids on a planar surface (solid curves). The MARTINI times in panel a are reported with their rescaled values (in which the simulation times are multiplied by a factor of 4 to address the known acceleration of MARTINI dynamics noted in the text.).

**Table 2:** Kolmogorov–Smirnov (KS) statistics comparing MARTINI + P_4_ (2nd and 3rd columns) and DPD-4 (4th and 5th columns) models with the All-Atom reference^63^ at selected times. In the KS statistics, *D* is defined as the maximum absolute difference between the empirical cumulative distribution functions of the two samples; here, *α* denotes the significance level, set to 0.05. *p*-Value indicates the significance of the agreement between the two distributions when *α* is greater than 0.05. The MARTINI times noted here have been multiplied by a factor of 4 as also reported in Fig. 5

Time	MARTINI *vs.* AA		DPD *vs.* AA	
	*D*	*p*-value	*D*	*p*-value
10 ps	0.355	0.003	0.045	0.578
100 ps	0.271	6.60 × 10^−5^	0.132	0.266
1 ns	0.131	0.266	0.102	0.578
4 ns	0.056	0.579	0.073	0.999

We also found that the nanoscale dynamics persist at longer time-and length-scales of our mesoscale DPD-4 and MARTINI + P_4_ lipid vesicle models. After obtaining the intermediate scattering function from the Fourier transform of the van Hove function,

8 *F*_s_(*q*,*t*) = ∫*G*_s_(*r⃑*,*t*)*e*^−*iq⃑*·*r⃑*^d*r⃑*,

where *q* = |*q⃑*|, as shown in Fig. 6, we found that both models capture the functional form of the experimentally justified Zilman–Granek stretched-exponential relaxation,^50^ though not yet the quantitative relaxation rate, as discussed further below. The comparison in Fig. 6 of the scattering functions at 4 ns obtained using MARTINI + P_4_ model shows similar performance as the DPD-4 model from the best Zilman–Granek model fits. Thus, both DPD-4 and MARTINI + P_4_ lipid parameters developed here can be used to construct models of vesicles of arbitrary size while capturing the functional form of the experimentally observed relaxation dynamics of SUVs, though not yet its absolute magnitude (Table 3).

**Fig. 6:**
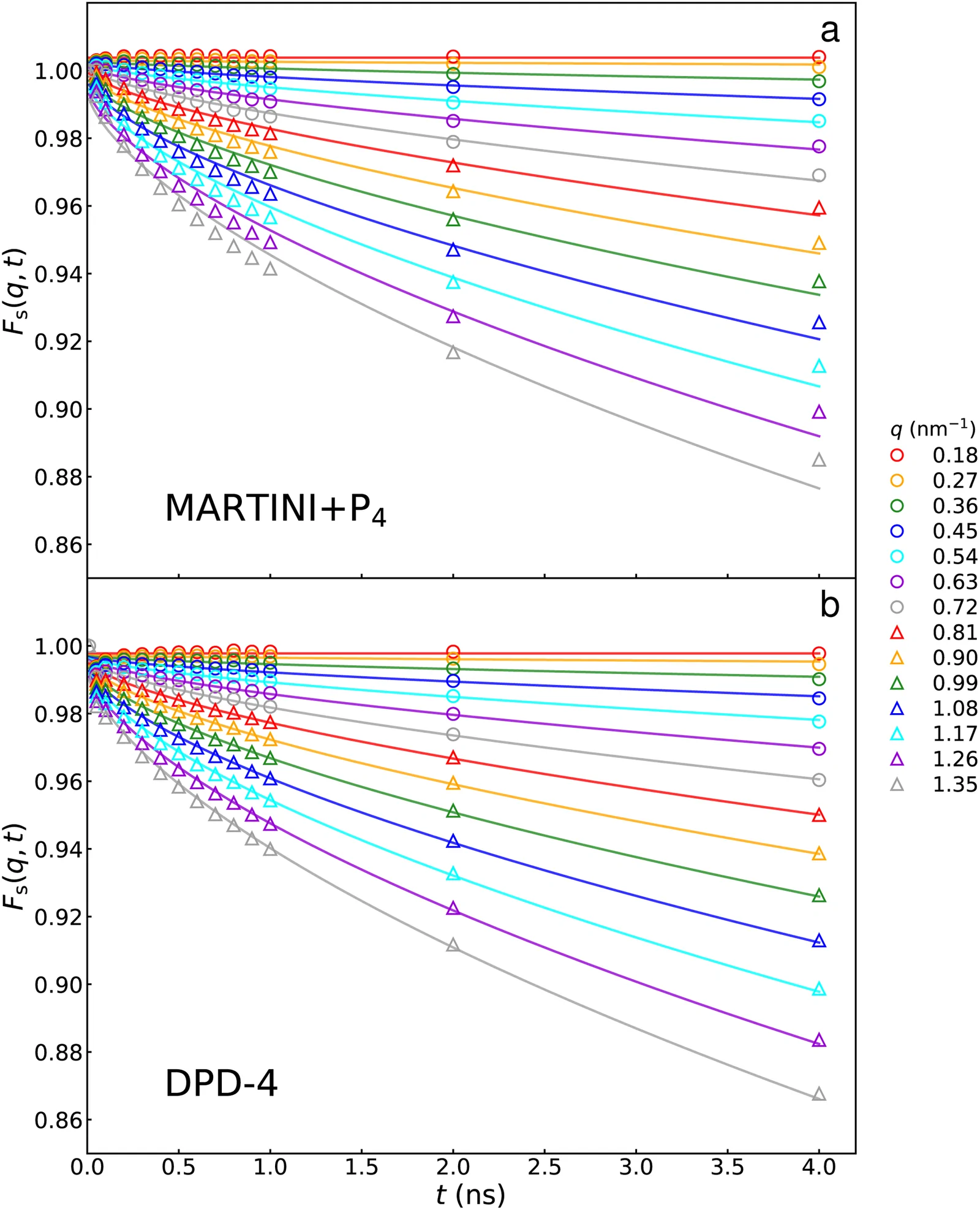
The intermediate scattering functions *F*_s_(*q*, *t*) of eqn (8) for a 60 nm vesicle (markers) obtained using MARTINI + P_4_ and DPD-4 simulations in panels (a) and (b), respectively, are compared to the best fits (solid lines) from the Zilman–Granek model over a range of wave numbers *q*, as listed in the legend. The MARTINI times in panel a have been scaled by multiplying by a factor of 4, as also noted in the caption of Fig. 5.

**Table 3:** The goodness of fit *R*^2^ for the Zilman–Granek fit of *F*_s_(*q*,*t*) = exp[−(*Γq*^3^*t*)^2/3^] for a single *Γ* to the data from MARTINI + P_4_ and DPD-4 simulations. The fitting parameters *Γ*_DPD_ ≈ 5.18 Å^3^ ns^−1^ and *Γ*_MARTINI_ ≈ 4.62 Å^3^ ns^−1^ after rescaling by a factor of 4. For an extended 150 ns window (Fig. 7), *Γ*_DPD_ ≈ 19.58 Å^3^ ns^−1^ and *Γ*_MARTINI_ ≈ 16.87 Å^3^ ns^−1^

*q* (nm^−1^)	4 ns window		150 ns window	
	*R*_MARTINI_^2^	*R*_DPD_^2^	*R*_MARTINI_^2^	*R*_DPD_^2^
0.18	−0.36	−0.16	0.80	0.64
0.27	0.33	0.49	0.82	0.91
0.36	0.84	0.83	0.83	0.96
0.45	0.96	0.93	0.86	0.97
0.54	0.98	0.97	0.88	0.98
0.63	0.99	0.98	0.91	0.99
0.72	0.99	0.98	0.93	0.99
0.81	0.99	0.98	0.95	0.99
0.90	0.99	0.99	0.97	0.99
0.99	0.98	0.99	0.98	0.99
1.08	0.98	0.99	0.99	0.99
1.17	0.98	0.99	0.99	0.99
1.26	0.98	0.99	0.99	0.99
1.35	0.98	0.99	0.99	0.99
Overall	0.99	0.99	0.97	0.99

In Table 3, the Zilman–Granek fits *F*_s_(*q*,*t*) = exp[−(*Γq*^3^*t*)^2/3^] yield a nearly *q*-independent pre-factor for the DPD model, with *Γ*_DPD_ ≈ 5.18 Å^3^ ns^−1^ and coefficients of determination *R*^2^ ≥ 0.94 for *q* ≥ 0.45 nm^−1^. This constancy across wave numbers indicates that the same stretched-exponential form governs the dynamics over the range relevant to the 4 ns comparison, supporting the reliability and internal consistency of the DPD parameters. The lower *R*^2^ values at small *q*, at *q* = 0.18 to 0.27 nm^−1^ in Table 3 for both DPD and MARTINI suggest the limitations of the models at large length scales. Fitting across the full range of wave numbers, rather than only where agreement is known to be good, allows us to identify this crossover. The low-*q* wave numbers correspond to wavelengths 2π/*q* ≈ 23–35 nm, comparable to the 60 nm vesicle's own radius, where low-order whole-vesicle shape (breathing/undulation) modes at the vesicle's own curvature scale—distinct from the rigid-body translation and rotation already removed prior to computing *G*_s_(*r*, *t*)—increasingly compete with the local, sub-vesicle membrane relaxation that the Zilman–Granek planar-membrane model was derived to describe. The *q*-dependence of the fit quality therefore maps out the length-scale range over which the planar Zilman–Granek description remains a good approximation for a finite, curved vesicle. Specifically, after applying a rescaling factor of 4, the MARTINI + P_4_ yields a pre-factor of *Γ*_MARTINI_ ≈ 4.62 Å^3^ ns^−1^, reflecting a similar relaxation time scale as DPD. Together with the statistics reported in Table 3, we thus find internal consistency of the representations, and correspondence in *q*, of both the DPD-4 and MARTINI + P_4_ dynamics. In turn, this internal consistency supports their transferability to vesicles of different sizes, independent of the quantitative agreement with experiment addressed below. The experimental *Γ* value for lipid vesicles is 20–26 Å^3^ ns^−1^,^50^ which means that both MARTINI and DPD models need further improvement to match experiments.

To address whether the weak low-*q* fits in the 4 ns window (Table 3) reflect an insufficient analysis window rather than a fundamental breakdown of the Zilman–Granek description, we reanalyzed both models over a much longer window (150 ns, with MARTINI times already rescaled by the standard factor of 4; Fig. 7), using the same single-shared-*Γ* fitting procedure. The low-*q* fits improve substantially: *R*^2^ at *q* = 0.18 rises from −0.16 to 0.64 (DPD-4) and from −0.36 to 0.80 (MARTINI + P_4_), and at *q* = 0.27 from 0.49 to 0.91 (DPD-4) and from 0.33 to 0.82 (MARTINI + P_4_), confirming that the 4 ns window was indeed too short for the low-*q*, long-wavelength modes to be well constrained. The extended-window fits also revise the fitted prefactor substantially, to *Γ*_DPD_ ≈ 19.58 Å^3^ ns^−1^ and *Γ*_MARTINI_ ≈ 16.87 Å^3^ ns^−1^—both markedly closer to the experimental value (20–26 Å^3^ ns^−1^)^50^ than the original 4 ns-window estimates. A modest single-*Γ*-vs-data mismatch remains visible for *q* ≤ 0.72 over this longer window, so we do not interpret the extended fit as a fully quantitative match to a single relaxation law across the entire accessible *q*-range; rather, it demonstrates that the short 4 ns window, not an intrinsic limitation of the low-*q* modes themselves, was the dominant factor behind the previously weak low-*q* agreement. As a further dynamical observable for the 12 nm vesicle, we compute the lateral (tangential) diffusion of phosphate head groups relative to the local vesicle surface, after removing overall vesicle rotation (Table S5 and Fig. S9 in the SI).

We report a workflow in the SI for constructing vesicles of any size. It can be used to model large complex mixtures of vesicles with other materials—*e.g.*, nanoparticles—relevant to applications such as drug transport. We have identified structural and dynamical measurables useful for developing and validating DPD models of varied lipid types: this includes, for example, the distribution of lipid tail end-to-end distances benchmarked by the structure of pre-packed All-Atom vesicles. It also includes global structural properties, such as the density profiles across and thickness of DMPC-lipid bilayers, relative to which the original parameters were validated.^17^ Our modified DPD lipid model can also produce the local packing of individual lipid tails within vesicles as a function of vesicle curvature. In capturing the finer-grained, molecular-level structural behavior within vesicles of 12 nm diameter, the model is thus well-suited to determine local structural transformations at nano-bio interfaces, as we have demonstrated in characterizing the size-dependent vesicle deformations upon insertion of nanoparticles.^30^ This structural fidelity at the curvature scales relevant to nanoparticle-vesicle contact supports the use of the validated DPD-4 parameters to predict nanoparticle wrapping and insertion thermodynamics, and could, for example, inform the design of nanoparticle surface chemistries in membrane-penetrating drug vectors.

## 4 Conclusions

The critical finding in this work is that a DPD-4 model with parameterization derived from MARTINI bead mappings and bonded terms (Sec. 2.3), exhibits structural and dynamical consistency with MARTINI, and structural consistency with corresponding All-Atom models of systems as complex as a lipid vesicle. Thus, the All-Atom and MARTINI benchmarking models can be used to structurally parameterize DPD vesicle systems so as to access similar length- and time-scales. The DPD model was also implemented on USUVs that are large enough to be interrogated experimentally while currently being too large to use other models. We did not benchmark this longer timescale behavior with an All-Atom simulation because doing so would be cost prohibitive, as reported herein. Instead, we found that the membrane dynamics in 60 nm SUVs follow the same Zilman–Granek stretched-exponential functional form observed in experiments, though the fitted relaxation prefactor *Γ* differs from the experimental value by a factor of ∼4 (Sec. 3.5), indicating that further parameterization is needed for quantitative agreement. Specifically, for the long-wavelength and low-*q* dynamics, the fits over an extended 150 ns window show a modest mismatch between the fitted *Γ* and the data at *q* ≤ 0.72 (Table 3 and Fig. 7). Thus we have found that a bottom-up approach can model large SUVs (12–60 nm). Extending this approach to giant unilamellar vesicles (GUVs) and, eventually, minimal whole-cell^70^ models remains an important long-term goal, but would require validating multicomponent, protein-containing membranes rather than single-lipid vesicles, and substantially longer length- and time-scales and coarser-scale dynamics well beyond the 12–60 nm µs^−1^ regime validated here.

**Fig. 7:**
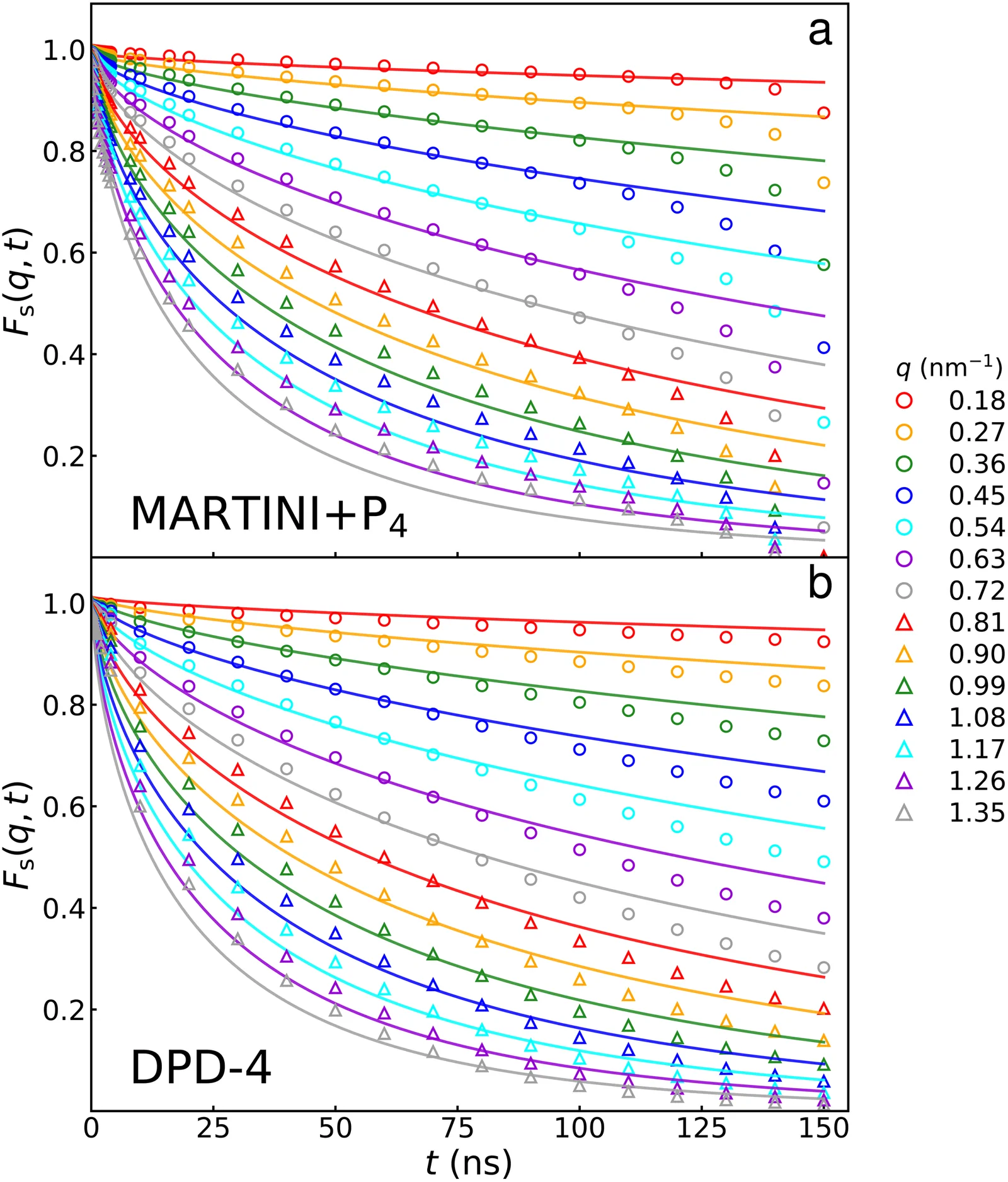
The intermediate scattering functions *F*_s_(*q*, *t*) of eqn (8) for a 60 nm vesicle (markers) shown in Fig. 6 are shown here for MARTINI+P4 and DPD-4 simulations in panels (a) and (b), respectively, over an extended 150 ns window. Both the fits (solid lines) and all computed data points (markers) are defined as in the earlier figure.

For this particular system, the structural consistency claimed in this mapping, particularly when performed through the decimation of a renormalization transformation is necessarily accurate at the longer scales that it retains. It also necessarily removes the shorter time scales that have been averaged over, and thus such CG models are inaccurate at short length scales while still being potentially accurate at longer length scales. The dynamical consistency that we found in these models is more difficult to prove rigorously because the resulting CG equations of motion necessarily involve stochastic terms. Nevertheless, coarse-graining the spatial variables effectively coarse-grains the time variables. That is, the short-time behavior is no longer accurate because the CG representation does not contain the small spatial variables. It can nevertheless be accurate at the longer times captured by the CG representation. In this sense, we have demonstrated that for this class of systems, both DPD and MARTINI representations are dynamically consistent. Thus, we have provided a reliable cross-scale validation and parameterization procedure for phospholipid bilayers and DMPC vesicles (12–60 nm). This suggests, but remains to be proved, that spatio-temporal properties of more complex membranes containing cholesterol or unsaturated lipids would also be accessible to these models as well as to newer CG models such as MARTINI3.

## Author contributions

Wandi Xu: data curation, formal analysis, investigation, methodology, resources, software, validation, visualization, writing – original draft (lead), writing – review and editing. Xingfei Wei: data curation, formal analysis, investigation, methodology, resources, software, supervision, validation, visualization, writing – original draft (lead), writing – review and editing. Gene Chong: data curation, formal analysis, investigation, methodology, resources, software, validation, visualization, writing – original draft (supporting), writing – review and editing (supporting). Udaya Dahal: data curation, formal analysis, investigation, methodology, resources, software, validation, visualization, writing – original draft (supporting), writing – review and editing (supporting). Qiang Cui: conceptualization (equal), formal analysis, funding acquisition, methodology, project administration, supervision (equal), writing – review and editing. Rigoberto Hernandez: conceptualization (equal), formal analysis, funding acquisition, methodology, project administration, supervision (equal), writing – review and editing (lead).

## Conflicts of interest

There are no conflicts to declare.

## Supplementary Material

RA-016-D6RA06719K-s001

## Data Availability

Input files for both GROMACS (MARTINI) and LAMMPS (All-Atom and DPD) simulations, together with the code necessary for reproducing our models, are available at https://github.com/rxhernandez/USUV. Supplementary information (SI): additional parameters and methods needed to fully specify the simutlations of the DPD, MARTINI and all-atom models; additional structural (radial distribution functions, density profiles, lipid end-to-end distance) and dynamical (van Hove function) observations not shown in the main text, including a convergence check using an independently extended 10 ns all-atom trajectory; and the DPD-3 model observations for the corresponding to those for the DPD models shown here. See DOI: https://doi.org/10.1039/d6ra06719k.
