# Supplementary material for: Structural and dynamical consistency in lipid vesicles across all-atom, MARTINI, and dissipative particle dynamics
Source: RSC Adv. 2026 Sep 8;16(43):53341–53. doi: 10.1039/d6ra06719k (PMC13551285; doi:10.1039/d6ra06719k)
Supplement: Supplementary file 1 [file RA-016-D6RA06719K-s001.zip › si.pdf]

Supporting Information for "Structural and Dynamical Consistency in Lipid Vesicles across All-Atom, MARTINI, and Dissipative Particle Dynamics"

Wandi Xu,† Xingfei Wei,† Gene Chong,[a]† Udaya Dahal[b],‡ Qiang Cui,‡ and Rigoberto Hernandez*,¶,§,‖

†*Department of Chemistry, Johns Hopkins University, Baltimore, Maryland 21218, USA*

‡*Department of Chemistry, Boston University, Boston, Massachusetts 02215, USA*

¶*Department of Chemistry, Johns Hopkins University, Baltimore, Maryland 21218, USA*

§*Department of Chemical & Biomolecular Engineering, Johns Hopkins University, Baltimore, Maryland 21218, USA*

‖*Department of Materials Science and Engineering, Johns Hopkins University, Baltimore, Maryland 21218, USA*

E-mail: r.hernandez@jhu.edu

[a]Present address: Springer Nature, Washington, D.C. 20005, USA

[b]Present address: Department of Physics and Engineering, Xavier University of Louisiana, New Orleans, LA 70125, USA

Contents

S1 Detailed Computational Methods **S3**

S1.1 Dissipative particle dynamics (DPD) Simulations S3

S1.2 Vesicle Models: Coarse-grained (CG) Mapping of DMPC Using MARTINI and DPD-4 . . . . S4

S1.3 Ratio of Inner and Outer Lipid Numbers S7

S2 Supplementary and Figures **S9**

S2.1 Radial distributions and correlation functions S9

S2.2 The van Hove functions S12

S2.3 Scattering function S13

S2.4 Lateral diffusion (12 nm vesicle) S13

S3 Supplementary for the DPD-3 model **S14**

S3.1 Simulation for the DPD-3 Model S14

S3.2 Parameter settings for DPD-3 . . . . S15

S3.3 Structure for the DPD-3 Model S17

References **S31**

S1 Detailed Computational Methods

S1.1 DPD Simulations

The DPD-4 model for the 1,2-dimyristoyl-sn-glycero-3-phosphocholine (DMPC) is described in the main paper following the model shown at the top of Figure S1 (for $N_m = 4$), where approximately 4 heavy atoms are CG into a single bead of a lipid model.[59] Based on previous work,[5] the DPD force field primarily depends on the repulsion parameter a_{ij} and the friction coefficient γ, as defined in the fluctuation–dissipation theorem:

$$\sigma^2 = 2\gamma k_B T \; . \tag{S1}$$

The simulations are conducted using reduced units. The pairwise interaction cutoff distance r_c is set to 1.0 in reduced distance units, which corresponds to 7.11 Å in the corresponding real units. The mass of each bead is also set to unity, and the time unit τ is determined to be 91.4 ps. For $N_m = 4$ particles, the friction coefficient is set to $\gamma = 4.5$. The bonded parameters are directly converted from the MARTINI force field.

In the DPD force field model, the primary interaction between two particles is repulsion. Specifically, the potential used to describe particle interactions consists of the Slater Coulomb potential for charged particles and the DPD potential for all particle types.[5] Notably the included electrostatic interactions allow for particle overlap. Based on previous studies,[5,30] the dielectric constant of the Slater potential is set to $\epsilon_r = 78.3$. The nonbonded interaction parameter for solvent beads is set to $a_{ij} = a_{ww} = 102$ in reduced energy units, which reproduces the compressibility of water at 300 K. For non-water particles, the values of a_{ij} are determined by the relation:

$$a_{ij} = a_{ww} + \bar{\chi}_{ij} \frac{N_m}{\lambda_m} \tag{S2}$$

where a_{ww} is the interaction parameter for water, $\bar{\chi}_{ij}$ is a scaling factor, N_m is the number of CG particles per bead, and λ_m is a system-dependent parameter. The resulting non-bonded

Table S1: DPD-4 nonbonded parameters a_{ij} between bead types—viz for i or j in (Q_0,Q_a,N_a,C, water)—as described in DPD-4 in Figure S1 top scheme.

a_{ij}	Q_0	Q_a	N_a	C	water
Q_0	102	98	98	121	84
Q_a		102	108	130	94
N_a			102	80	108
C				102	130
water					102

Table S2: DPD-4 bond-stretch and angle-bend parameters between bead numbers, as described by DPD-4 in Figure S1 top scheme. Here, "1-2" denotes the bond between bead 1 and bead 2, "1-2-3" denotes the angle between bonds, and "1-2" and "2-3" with central bead 2 as the vertex of the angle.

Bond type	Bond Stretch (DMPC Lipids)	$K_{ij}(k_BT)$	$r_{eq}(r_c)$
1	1-2, 2-3<br>4-5, 5-6, 6-7, 7-8<br>4-9, 9-10, 10-11, 11-12	127	0.66
2	3-4	127	0.52
**Bond type**	**Angle Bend (DMPC Lipids)**	$K_{ij}(k_BT)$	$\theta_{eq}(deg)$
1	2-3-5, 3-5-6, 5-6-7, 6-7-8<br>4-9-10, 9-10-11, 10-11-12	5.0	180.0
2	2-3-4	5.0	120.0

interaction parameters are shown in Table S1. The bond-stretch, and angle-bend interactions are translated from the MARTINI values shown in Table S2.

S1.2 Vesicle Models: CG Mapping of DMPC Using MARTINI and DPD-4

The methods for constructing the vesicles using all-atom, DPD-4 and MARTINI representations are summarized briefly in the main paper. As the workflow for such a construction and its validation using a DPD-4 model is less standard, we provide a more detailed description here. Note that in this work, we explicitly constructed 620-, 1500-, 3016-, and 4084-DMPC lipid vesicle models, but the workflows are the same regardless of the size.

The workflow can be described through the following steps:

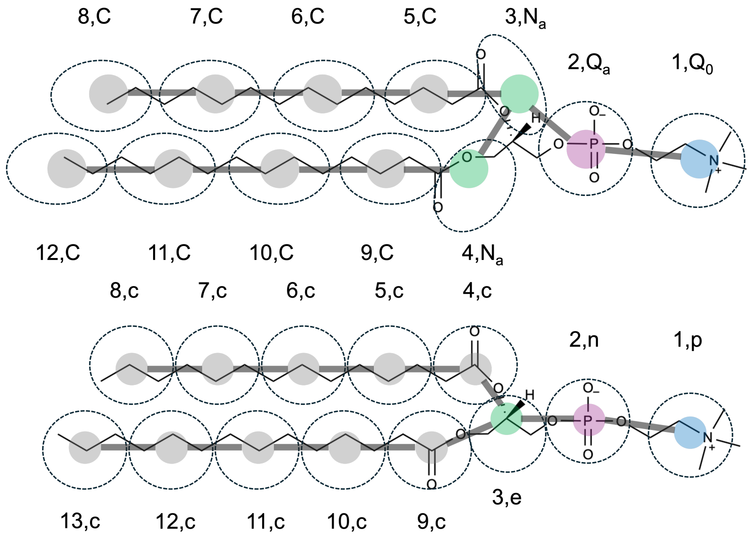

Figure S1: Two schemes for CG the DMPC lipid model are shown overlayed on the all-atom structure. Each bead is labeled by a bead number and a bead type. In the CG DPD-4 (**Top**) primarily reported in the main text, each DPD bead represents approximately **4** heavy atoms: positively charged quaternary ammonium groups ($1Q_0$), negatively charged phosphate groups ($2Q_a$), glycerol groups ($3N_a$, $4N_a$) ester-linked to the lipid tails, and CH_2 and CH_3 groups (C) in the lipid tails. In the alternative CG DPD-3 (**bottom**) reported in this SI, each DPD bead represents approximately **3** heavy atoms: positively charged quaternary ammonium groups (p), negatively charged phosphate groups (n), ester-linked glycerol groups to lipid tails (e), and methylene (CH_2) and methyl (CH_3) groups (c) in the lipid tails. DPD-3 was employed in our earlier work,[30] and it is also reported in this Supporting Information (SI) in comparison to BMW, POL water, and MARTINI.

(1) The CG mapping of DMPC lipids follows the methodology of the MARTINI force field, where four heavy atoms are represented as a single CG bead, as shown at the top of Figure S1. A useful reference for constructing the DPD-4 representation of 1,2-dipalmitoyl-sn-glycero-3-phosphocholine (DPPC) can be found in the established MARTINI model. Following the same scheme, we first construct the MARTINI model and then develop the DPD-4 model accordingly.[9] As DMPC contains shorter acyl chains (14 carbons per tail) than DPPC (16 carbons per tail), adjustments are necessary to account for the reduced chain length, particularly in the definition of the terminal beads.

(2) The headgroup of DMPC is represented by three beads: a positively charged Q_0 bead for the ammonium group, a negatively charged Q_a bead for the phosphate group, and two

polar N_a beads for the glycerol backbone. These beads capture the hydrophilic and charged nature of the lipid's polar regions, ensuring accurate representation of its headgroup.

(3) For the tails, the MARTINI mapping for DMPC follows the same approach as for DPPC, where each bead typically represents four carbon atoms and is classified as a non-polar C bead. However, due to the shorter tail length of DMPC, the final two beads (C8 and C12) each represent two carbon atoms instead of four. This adjustment maintains consistency with the MARTINI model while accurately reflecting the reduced tail length of DMPC. Thus, the mapping framework is nearly identical for both lipids, differing only in the representation of the terminal beads.

(4) The MARTINI mapping serves as the foundation for constructing the DPD-4 model of DMPC. One needs only to modify particle sizes and interaction potentials to adapt to the DPD framework. The ammonium (Q_0) and phosphate (Q_a) beads retain their electrostatic properties, modeled through appropriate repulsion parameters in the DPD force field. For the tail chains, the terminal beads (C_8 and C_{12}) are represented with half the effective mass of the regular beads to account for their reduced carbon content. This adjustment ensures that the mass distribution and dynamic behavior of DMPC are accurately represented in the DPD-4 simulations.

(5) To build a 12 nm vesicle, our all-atom model is constructed based on the expected density of the lipids in the bilayer while correcting for the finite-size effects due to the curvature for a given diameter. The all-atom models of DMPC and TIP3P water molecules were packed using MATLAB and LAMMPS.[53] Initially the DMPC lipids were randomly distributed, forming a spherical vesicle, then it was solvated in a water environment. All-atom molecular dynamics (MD) simulations were propagated using LAMMPS[53] with the CHARMM36 force field.[52] The models were equilibrated constraining NPT at constant pressure 1 atm and temperature 300 K. The timestep was set to 2 fs. Both the all-atom and DPD-4 models for the 12 nm vesicle equilibrated to aspherical structures displaying small planar regions of the vesicle surface (Fig. 1a in the main paper). Such deviations from

a perfect spherical vesicle structure is attributed to the high bending resistance of smaller vesicles with high surface curvatures.[64,65]

S1.3 Ratio of Inner and Outer Lipid Numbers

The ratio for the number of lipids of the inner and outer shells of any given model vesicle is expected to be the ratio of the radii of the shells assuming perfect spheres based on geometric arguments. Specifically, for a vesicle with a given diameter, the outer and inner lipid numbers, N_{outer} and N_{inner}, can be determined by considering the volumes of the respective spherical shells. Each shell has a thickness of 2 nm, and the lipid volume per molecule is taken as $1.32\,\text{nm}^3$ (area per lipid $0.66\,\text{nm}^2$ multiplied by the bilayer thickness of $2\,\text{nm}$). The lipid number in the outer layer is calculated as:

$$N_{\text{outer}} = \frac{V_{r_1} - V_{r_2}}{1.32\,\text{nm}^3}, \tag{S3}$$

where $V_{r_1} = \frac{4}{3}\pi r_1^3$ and $V_{r_2} = \frac{4}{3}\pi r_2^3$ are the volumes of spheres with radii r_1 and $r_2 = r_1 - 2\,\text{nm}$, respectively. Similarly, the inner lipid number is given by:

$$N_{\text{inner}} = \frac{V_{r_2} - V_{r_3}}{1.32\,\text{nm}^3}, \tag{S4}$$

where V_{r_2} and V_{r_3} correspond to the inner sphere radii r_2 and $r_3 = r_2 - 2\,\text{nm} = r_1 - 4\,\text{nm}$, respectively. The relationship between the number of lipids in the outer and inner layer follows the equation:

$$\frac{N_{\text{outer}}}{N_{\text{inner}}} = \frac{r^3 - (r - 2\,\text{nm})^3}{(r - 2\,\text{nm})^3 - (r - 4\,\text{nm})^3}, \tag{S5}$$

where r represents the vesicle's outer radius. Although our simulation model is not explicitly constructed based on this geometric relationship, the resulting equilibrated structures exhibit lipid number ratios in good agreement with Eq. S5 as shown in Fig. 2 in the main paper and Fig. S2 in this SI.

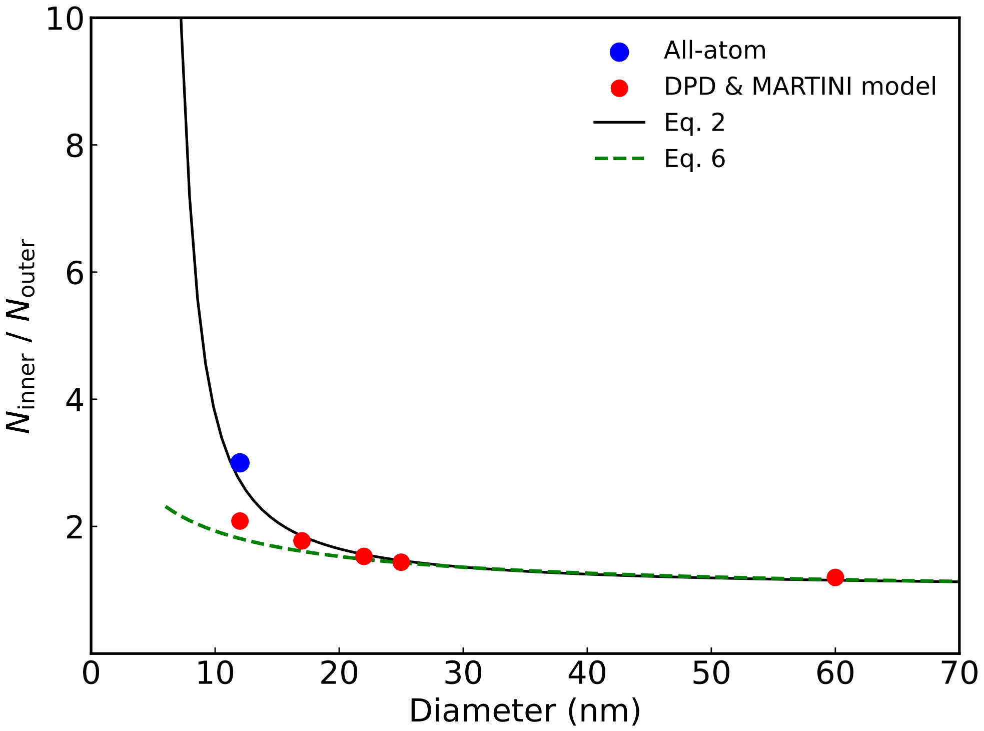

Figure S2: The correspondence between outer and inner lipid numbers—N_{outer} and N_{inner} in ultrasmall unilamellar vesicles (USUVs). The vesicle membranes containing total lipid counts of 620, 1500, 3016, and 4084 correspond to vesicle diameters of 12, 17, 22, and 25 nm, respectively. The DPD-4 and MARTINI+P_4 CG models (in red) resulted in the same numbers, and are compared to the all-atom 12 nm model (in blue). The theoretical result from Eq. 2—viz. Eq. S5—is shown in black. The theoretical ratio[35] (green dashed curve) of Eq. 6 in the main paper obtained from large vesicles is shown for comparison. This can support Figure 2 in the main text.

Table S3: Quantitative comparison of the 12 nm vesicle density profiles shown in Fig. 1 of the main text (all-atom (AA), MARTINI+P_4, and DPD-4), for each functional group. The ammonium and phosphate head-group profiles are bimodal (inner- and outer-leaflet peaks); their separation (Layer sep.) is a proxy for the head-group-to-head-group bilayer thickness. The carbon tail profile is unimodal (a single peak spanning both leaflets' tails, listed under Inner/Outer peak as a merged column), for which the peak position and width instead characterize the hydrophobic core. Peak FWHM is computed locally around each identified peak (not across the full profile, which would otherwise conflate the two head-group peaks for a bimodal channel). D is the Kolmogorov–Smirnov statistic between each CG model's density profile and the all-atom reference, after normalizing each profile to a probability density ($D = 0$ indicates identical distributions). The all-atom reference is only available for the 12 nm vesicle.

Group	Model	Inner (nm)	Outer (nm)	Layer sep. (nm)	FWHM (nm)	D vs. AA
Ammonium (N)	AA	2.59	5.95	3.36	0.83	—
	MARTINI+P_4	2.35	5.94	3.59	0.64	0.280
	DPD-4	2.24	6.17	3.93	0.94	0.195
Phosphate (P)	AA	2.75	5.82	3.08	0.72	—
	MARTINI+P_4	2.31	5.89	3.59	0.57	0.352
	DPD-4	2.60	5.93	3.34	0.63	0.162
Carbon (C)	AA	4.59		—	2.24	—
	MARTINI+P_4	4.75		—	2.37	0.039
	DPD-4	4.38		—	2.00	0.100

S2 Supplementary and Figures

S2.1 Radial distributions and correlation functions

Table S3 quantifies the visual agreement among the three models shown in Fig. 1 of the main text. For the ammonium and phosphate head groups, DPD-4 is closer to the all-atom reference ($D = 0.195$ and 0.162, respectively) than MARTINI+P_4 ($D = 0.280$ and 0.352). For the carbon tail region, the opposite is true: MARTINI+P_4 is closer to the all-atom reference ($D = 0.039$) than DPD-4 ($D = 0.100$). All three models place the inner-leaflet head-group peaks within about 0.5 nm of the all-atom reference (2.59 nm for ammonium, 2.75 nm for phosphate), and the outer-leaflet peaks agree even more closely (within 0.2–0.3 nm of 5.95 nm and 5.82 nm, respectively), while the carbon peak position differs by less than 0.4 nm across

all three models (4.38–4.75 nm vs. 4.59 nm for all-atom). The inner-to-outer layer separation is 3.1–3.4 nm for the all-atom reference, and is somewhat larger for both CG models (3.3–3.9 nm). This may partly reflect the fact that the DPD-4/MARTINI+P_4 topology is adapted from a DPPC (16 carbons per tail) reference by shortening only the terminal tail beads to represent the shorter DMPC (14 carbons per tail) chain (Sec. S1.2), rather than being re-derived natively for DMPC.

Reviewer 2 noted that Table S3 and Fig. 1 of the main text report single-trajectory point estimates with no uncertainty quantification, and asked for additional structural and dynamical observables such as bilayer thickness distributions and lateral diffusion coefficients. Although multiple independent trajectories were generated for each vesicle size and model, only one representative trajectory was selected for this level of detailed analysis (Sec. 2.3); we therefore cannot report genuine inter-replica error bars here, and instead estimate uncertainty by block-averaging within the selected trajectory for each model (5 contiguous time blocks per model).

Figure S5 shows the same 12 nm density profiles as Fig. 1 of the main text, now with a shaded band indicating the block-to-block standard error at each radial bin. The bands are narrow relative to the peak heights, indicating that the profiles are statistically stable within each single trajectory.

Table S4 reports the block-to-block distribution (mean ± standard error over 5 blocks) of two thickness measures already introduced in Table S3: the inner-to-outer head-group layer separation, and the hydrophobic-core carbon peak FWHM, for all three models.

Figure S3 presents the radial distribution functions between lipid functional groups and the vesicle vesicle center of mass (COM) for vesicles of various sizes in DPD simulations. For a 12-nm vesicle composed of 620 lipids, the peak for carbon groups is at 4.25 nm, ranging from 2.70 to 5.80 nm. The ammonium groups has two peaks, the first peak is at 2.2 nm, and the second peak is at 6.27nm. The phosphate groups exhibit similar peaks are at 2.75nm and 6.0nm. The inner layer contains an average of 201 lipids, with a surface density of 0.66

Table S4: Block-to-block distribution (mean ± standard error, 5 blocks) of the inner-to-outer head-group layer separation and the hydrophobic-core carbon peak FWHM for the 12 nm vesicle, for all three models. These are the same point quantities reported in Table S3, now with a block-averaged uncertainty estimate.

Model	Quantity	Value (nm)
All-atom	Layer separation (ammonium)	3.31 ± 0.03
	Layer separation (phosphate)	3.04 ± 0.03
	Hydrophobic core FWHM (carbon)	2.18 ± 0.01
DPD-4	Layer separation (ammonium)	3.90 ± 0.06
	Layer separation (phosphate)	3.35 ± 0.03
	Hydrophobic core FWHM (carbon)	1.99 ± 0.00
MARTINI+P_4	Layer separation (ammonium)	3.71 ± 0.07
	Layer separation (phosphate)	3.67 ± 0.05
	Hydrophobic core FWHM (carbon)	2.26 ± 0.02

nm^2 per lipid. For a 17-nm vesicle composed of 1500 lipids, the peak for carbon groups is at 6.27nm, ranging from 4.18 to 7.85 nm. The ammonium groups have two peaks: the first peak is at 3.92 nm, and the second peak is at 8.25 nm. The phosphate groups exhibit similar peaks, located at 4.23 nm and 8.01 nm. The inner layer contains an average of 541 lipids, with a surface density of 0.66 nm^2 per lipid. For a 22-nm vesicle composed of 3016 lipids, the peak for carbon groups is at 8.62 nm, ranging from 6.10 to 10.5 nm. The ammonium groups have two peaks: the first peak is at 6.12 nm, and the second peak is at 10.5 nm. The phosphate groups exhibit similar peaks, located at 6.44 nm and 10.3 nm. The inner layer contains an average of 1193 lipids, with a surface density of 0.66 nm^2 per lipid. For a 25-nm vesicle composed of 4084 lipids, the peak for carbon groups is at 9.85 nm, ranging from 7.12 to 12.20 nm. The ammonium groups have two peaks: the first peak is at 7.58.0 nm, and the second peak is at 12.10 nm. The phosphate groups exhibit similar peaks, located at 7.86nm and 11.75 nm. The inner layer contains an average of 1676 lipids, with a surface density of 0.66 nm^2 per lipid.

To further validate the structural consistency of the coarse-grained models, we computed the site-site radial distribution function $g(r)$ between phosphate head groups in a 12 nm vesicle. For the all-atom model, the phosphorus atom (P) was used as the reference site;

for the MARTINI and DPD models, the PO4 bead served as the equivalent site. The $g(r)$ was calculated using the `InterRDF` function in MDAnalysis with a bin width of 0.25 Å and a cutoff of 50 Å. All three models reproduce the primary and secondary coordination peaks near 5 Å and 10 Å, confirming that the essential non-bonded two-body lipid correlations are captured across resolution levels (Figure S4). The slightly sharper and shifted first peak in MARTINI (∼4 Å) reflects the reduced configurational degrees of freedom in the coarse-grained representation, while the broader features in DPD are consistent with its soft interaction potential.

To confirm that the 5 ns production window reported in the all-atom simulations in the main text is sufficient to capture converged structural statistics, the 12 nm all-atom vesicle was propagated by an additional 10 ns of production sampling, starting from the same equilibrated configuration from the ends of the original 5 ns runs. Figure S6 shows the comparison between the original 5 ns run and the extended 10 ns run for the radial distribution functions of the distance between the vesicle COM and the ammonium, phosphate, and carbon functional groups. Figure S7 makes the same comparison for the lipid tail end-to-end distance distributions. In both cases, the extended-simulation distributions closely align with the original 5 ns results, and confirm that the all-atom production window reported throughout the main text is sufficient for converged structural statistics.

S2.2 The van Hove functions

Figure S8 illustrates the van Hove function $G_\mathrm{s}(r,t)$ for lipid tail beads in the trajectory of the 60 nm vesicle DPD-4 and MARTINI+P_4 model in comparison to the all-atom simulations shown above. In the Figure S8, t corresponds to a fixed time interval. These figures report the probability that lipid tail beads move over different time steps, ranging from 10 ps to 4 ns. To facilitate comparison, we converted the original data from Ref. 63, where the probability density $P_t(r)$ (in nm^{-1}) was presented, into the probability function $G_\mathrm{s}(r,t)$.

S2.3 Scattering function

We report DPD-4 model and MARTINI+P_4 simulations only to 4 ns to obtain the scattering functions shown in Figure 6 in the main text. This choice of the maximum time was long enough to characterize the decay while avoiding needlessly long simulations. We compared them to the Zilman-Granek model over the same 4 ns timeframe. The DPD-4 model gives rise to good agreement with the functional form of the Zilman-Granek model. However, the fits to the MARTINI 60 nm vesicle model are not nearly in as good agreement with the Zilman-Granek model.

S2.4 Lateral diffusion (12 nm vesicle)

The mean-squared displacement (MSD) measures how far, on average, a particle has moved from its starting position after a time lag t; for ordinary (Fickian) diffusion in d dimensions, $\mathrm{MSD}(t) = 2dDt$, where D is the diffusion coefficient. For a curved vesicle, we are interested specifically in *lateral* (in-plane) diffusion, i.e., motion tangential to the local membrane surface, as distinct from radial motion (thickness fluctuation). We therefore track the phosphate head-group bead (all-atom: the raw phosphorus atom) relative to the vesicle center of mass, and decompose its displacement at each time lag into a component radial to the local vesicle surface and a component tangential to it, after first removing the vesicle's own rigid-body rotation frame-by-frame ($d = 2$ for this tangential-plane motion, so $\mathrm{MSD}_{\parallel}(t) = 4D_{\parallel}t$ for ordinary diffusion). The mean-squared tangential displacement, $\mathrm{MSD}_{\parallel}(t)$, is shown on log–log axes in Figure S9 for all three models, each evaluated on the same common time window (0.4–10 ns, the range over which all-atom, DPD-4, and MARTINI+P_4 trajectories are all available). All three curves are consistent with a power law, $\mathrm{MSD}_{\parallel} \sim t^{\alpha}$, with a similar sub-diffusive exponent α (the local slope of $\log \mathrm{MSD}_{\parallel}$ vs. $\log t$) across all three models: $\alpha \approx 0.80$ for the all-atom reference versus $\alpha \approx 0.55$–0.6 for the two CG models, indicating that all three retain sub-diffusive character—not yet ordinary (Fickian) diffusion—over this window. Because none of the models have reached the linear (Fickian) regime here, the lat-

Table S5: Apparent lateral diffusion coefficients $D_\parallel$ (from a local linear fit to the later half of each window) and anomalous-diffusion exponent α (local slope of $\log \mathrm{MSD}_\parallel$ vs. $\log t$) for the 12 nm vesicle, all three evaluated on the same common time window (0.4–10 ns) for direct comparability; for MARTINI+P$_4$, this window is quoted after the standard 4× dynamics rescaling applied throughout this work. All three models give $\alpha < 1$, indicating that the accessible trajectory window has not yet reached the long-time linear (Fickian) regime for this observable, so $D_\parallel$ should be read as an apparent, short-time value rather than an asymptotic diffusion coefficient.

Model	$D_\parallel$ (nm^2/ns)	α	Window (ns)
All-atom	0.0270 ± 0.0015	0.80	0.4–10
DPD-4	0.0232 ± 0.0001	0.55	0.4–10
MARTINI+P$_4$	0.0199 ± 0.0003	0.59	0.4–10

eral diffusion coefficients extracted from a local linear fit (Table S5) are *apparent, short-time* values, not converged asymptotic diffusion constants. With this caveat, all three models, now evaluated on the identical time window, give closely matching apparent diffusion coefficients ($D_\parallel \approx$ 0.020–0.027 nm^2/ns), indicating consistent sub-diffusive lateral dynamics across all-atom, DPD-4, and MARTINI+P$_4$ resolutions.

S3 Supplementary for the DPD-3 model

S3.1 Simulation for the DPD-3 Model

Figure S10 illustrates the self-assembly process of a vesicle from a planar membrane composed of 2750 lipids at 0, 50,000, and 100,000 timesteps. The images depict the dynamic transition of lipids from a planar structure to a vesicle over time, driven by the hydrophobic nature of the phospholipid bilayer in solution. This transition highlights the formation of the vesicle, showcasing the dynamic process of vesicle assembly. Figure S11 shows the representative structure of the 60 nm DPD-3 vesicle and its cross section view.

S3.2 Parameter settings for DPD-3

DPD-3 simulations were performed using bottom of Figure S1 in which approximately three heavy atoms are mapped into a single CG bead for the lipid model.[5] Reduced units were used with the pairwise-interaction cutoff distance r_c setting to 1.0, in reduced distance units, which is equivalent to 6.46 Å in real units.[17] The mass of each bead and the time unit τ were also set to unity. The energy unit was set to $\varepsilon = k_B T$(at $T = 300K$), which led to the reduced temperature $T^* = k_B T/\varepsilon = 1$. Harmonic potentials were used to describe both the bond stretching between two beads i and j, and the angle bending between three beads i, j and k around the equilibrium bond distances and angle sizes, respectively. They were calculated in the form of $U_{bond} = K_{ij}(r_{ij} - r_{eq})^2$ and $U_{angle} = K_{ijk}(\theta_{ijk} - \theta_{eq})^2$, respectively. For nonbonded interactions, the DPD conservative force F_{ij}^C in Eq. S6 and an electrostatic force F_{ij}^E in Eq. S7[60] were applied together with the random force[13] plus the dissipative force,[13] which was implemented by the DPD thermostat in the LAMMPS package.[53] For the electrostatic force, charged beads were assigned a +1 or -1 charge, and D was set to 0.65, as previously described.[60] The relative permittivity of the water solvent is $\varepsilon_r = 78.3$ and ε_0 is the vacuum permittivity.

The conservative and electrostatic forces in the DPD-3 model were calculated as following:[11–13]

$$F_{ij}^C = \begin{cases} a_{ij}(1 - r_{ij}/r_c)\hat{r}_{ij}, & r_{ij} < r_c \\ 0 & r_{ij} \geq r_c \end{cases}, \tag{S6}$$

$$F_{ij}^E = \frac{q_i q_j}{4\pi\varepsilon_r\varepsilon_0} \begin{cases} \frac{1}{r_{ij}^2}\sin^6(\frac{2\pi r_{ij}}{4D})\hat{r}_{ij}, & r_{ij} < D \\ \frac{1}{r_{ij}^2}\hat{r}_{ij} & D \leq r_{ij} < r_c \end{cases}, \tag{S7}$$

where the parameters, viz. — D, a_{ij}, r_{ij}, r_c — have already been specified in this appendix.

We used a bead density of $\rho = 3$ for all simulations, i.e. the total number of DPD beads in the system was three times the box volume (in units of r_c^3). In the DMPC model, three

heavy atoms were thereby CG to one DPD bead and the bead volume was 90 Å^3, which resulted in a lipid density of 4.8 molecules/nm^2 on a 4 nm spherical NP shell that agreed with experiments.[62] The nonbonded interaction parameter between solvent beads was set to $a_{ij} = 25$ in reduced energy units, which can reproduce the compressibility of water at 300 K. The parameters used for nonbonded, bond-stretch, and angle-bend interactions are presented in Table S6 and Table S7, and system sizes are presented in Table S8. The size of the simulation boxes for each of the vesicles was not optimized in Table S8 to achieve some target efficiency, but rather were constructed to obtain stable equilibrated structures for the sake of comparing the properties of the vesicles. Consequently, the systems with similar number of lipids and vesicle size are not all of the same dimensions, and the remaining volume was filled by a different number of solvent particles. In the case of the 25 nm vesicles, for example, the MARTINI and DPD-3 systems have edge lengths of 323 Å and 452.2 Å. That accounts for a factor of approximately 2.7 in volume which would require the MARTINI model to have about 2.2M beads to match the DPD-3 model box rather than the 811K required in the reported MARTINI model box. When taking this into account, the DPD-3 model does have both a reduction in the required number of beads and in the cost of the integration for a given vesicle size. This scaling allowed us to use DPD-3 for the much larger 60 nm vesicles with the computational resources that were available to us.

In the first step, we prepared planar membranes with equal number of lipids in the upper and lower leaflets. All of the surroundings, including the regions around the hydrophobic edges, were filled with water using Packmol.[61] We then ran ten simulations for each condition. The number of lipids in the inner layer and outer layer of a vesicle, and the number of water molecules inside the vesicle were counted from the last frame of each vesicle self-assembly simulation, which was averaged across all simulations. During these simulations, we found that the initially-planer membranes can self-assemble into vesicles through DPD simulations. This process generally occurs on the time-scale of hundreds of nanoseconds to microseconds,[16] which is generally not accessible through all-atom MD simulations.

Table S6: DPD-3 nonbonded parameters a_{ij} between bead types—viz. for i or j in (p, n, e, c, water)—as described in DPD-3 Figure S1 bottom scheme

a_{ij}	p	n	e	c	water
p	35	35	35	80	25
n		35	35	80	25
e			35	80	25
c				80	25
water					25

.

Table S7: DPD-3 bond-stretch and angle-bend parameters between bead numbers as specified by DPD-3 in Figure S1 bottom scheme. Here, "1-2" denotes the bond between bead 1 and bead 2, "1-2-3" denotes the angle between bonds, and "1-2" and "2-3" with central bead 2 as the vertex of the angle.

Bond type	Bond Stretch (DMPC Lipids)	$K_{ij}(k_BT)$	$r_{eq}(r_c)$
1	1-2, 2-3<br>3-4, 4-5, 5-6, 6-7, 7-8<br>3-9, 9-10, 10-11, 11-12, 12-13	100	0.7
**Bond type**	**Angle Bend (DMPC Lipids)**	$K_{ij}(k_BT)$	$\theta_{eq}(deg)$
1	4-5-6, 5-6-7, 6-7-8<br>9-10-11, 10-11-12, 11-12-13	6.0	180.0
2	2-3-4, 2-3-9, 3-4-5, 3-9-10, 4-3-9	3.0	120.0
3	1-2-3	1.0	180.0

S3.3 Structure for the DPD-3 Model

Figure S12 compares the radial distribution functions between different functional groups and the vesicle center of mass for the 12-nm vesicle in DPD-3, BMW-MARTINI, and all-atom simulations. For ammonium groups, the distance at half-maximum after the first peak, the effective inner radius, is approximately 2.5 nm, the second peak is at around 5.8 nm and density is 0.65 nm^2 per lipid. For phosphate groups, the distance at half-maximum after the first peak is around 2.5 nm, the second peak is approximately 5.6 nm. For carbon atoms, the distance at half-maximum after the first peak is starting from 2.0 nm and extending to around 6.0 nm.

Figure S13 presents the radial distribution functions between lipid functional groups and the vesicle COM for vesicles of various sizes in DPD-3 simulations. For a 12-nm vesicle

Table S8: Total sizes in terms of number of atoms or particles of the systems—including solvent—selected in this work. This table is to provide more details for our simulation models in the methods section in the main text.

All-Atom	Box size (Å^3)	Total atoms
640 lipids, 12 nm	180 × 180 × 180	580,603
MARTINI+P_4	Box size (Å^3)	Total beads
620 lipids, 12nm vesicle	184× 184 × 184	48,034
1500 lipids, 17nm vesicle	216 × 216 × 216	79,859
3016 lipids, 22 nm vesicle	264 × 264 × 264	148,710
4000 lipids, 25nm vesicle	323 × 323 × 323	206,148
27848 lipids, 60 nm vesicle	700 × 700 × 700	2,853,718
DPD-4	Box size (r_c^3; $\AA^3$)	Total beads
620 lipids, 12 nm vesicle	25.8 × 25.8 × 25.8; 184 × 184 × 184	48,034
1500 lipids, 17 nm vesicle	30.4 × 30.4 × 30.4; 216 × 216 × 216	79,859
3016 lipids, 22 nm vesicle	37.1× 37.1 × 37.1; 264 × 264 × 264	148,710
4084 lipids, 25 nm vesicle	41.4 × 41.4 × 41.4; 294 × 294 × 294	206,148
27848 lipids, 60 nm vesicle	108 × 98.5 × 98.5; 700× 700 × 700	2,853,718

composed of 576 lipids, the distance at half-maximum after the first peak for ammonium groups is 2.73 nm, which represents the effective inner radius. The average number of lipids in the inner layer of the 12-nm vesicle is 143.7, with a density of 0.65 nm^2 per lipid. For a 17-nm vesicle composed of 1500 lipids, the distance at half-maximum after the first peak is 5.18 nm, with an average of 518.8 lipids in the inner layer and a density of 0.65 nm^2 per lipid. For a 22-nm vesicle composed of 2750 lipids, the distance at half-maximum after the first peak is 7.47 nm, with an average of 1061.0 lipids in the inner layer and a density of 0.66 nm^2 per lipid. For a 25-nm vesicle composed of 4000 lipids, the distance at half-maximum after the first peak is 9.19 nm, with an average of 1613.9 lipids in the inner layer and a density of 0.66 nm^2 per lipid.

Figure S14 shows the distributions of lipid tail end-to-end distances, in the inner and outer layers of 12 nm, 17 nm and 25 nm diameter DPD-3 vesicles. Figure S15 shows the

van Hove function $G_\mathrm{s}(r,t)$ comparison between all-atom and the DPD-3 model for the 60 nm vesicle. The scattering function for the DPD-3 model is also plotted in Figure S16.

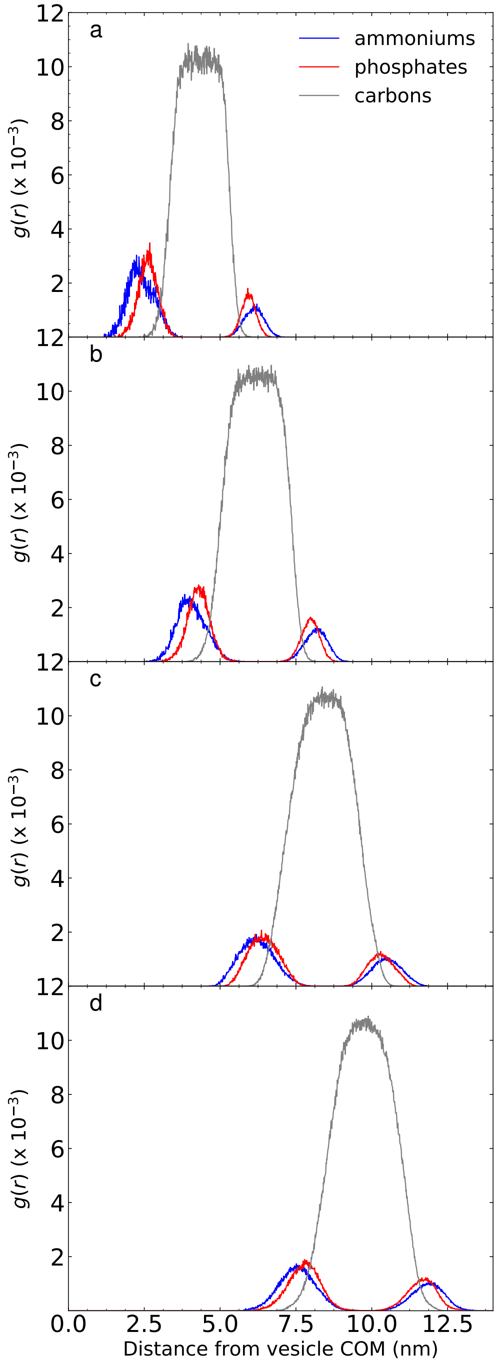

Figure S3: Radial distribution functions between lipid functional groups and the vesicle COM for different sized vesicles using the DPD-4 model for diameters including and beyond the 12 nm vesicle shown in Figure 1: a) ∼12-nm (640 lipids), b) ∼17-nm (1500 lipids), c) ∼22-nm (3016 lipids), and d) ∼25-nm (4084 lipids).

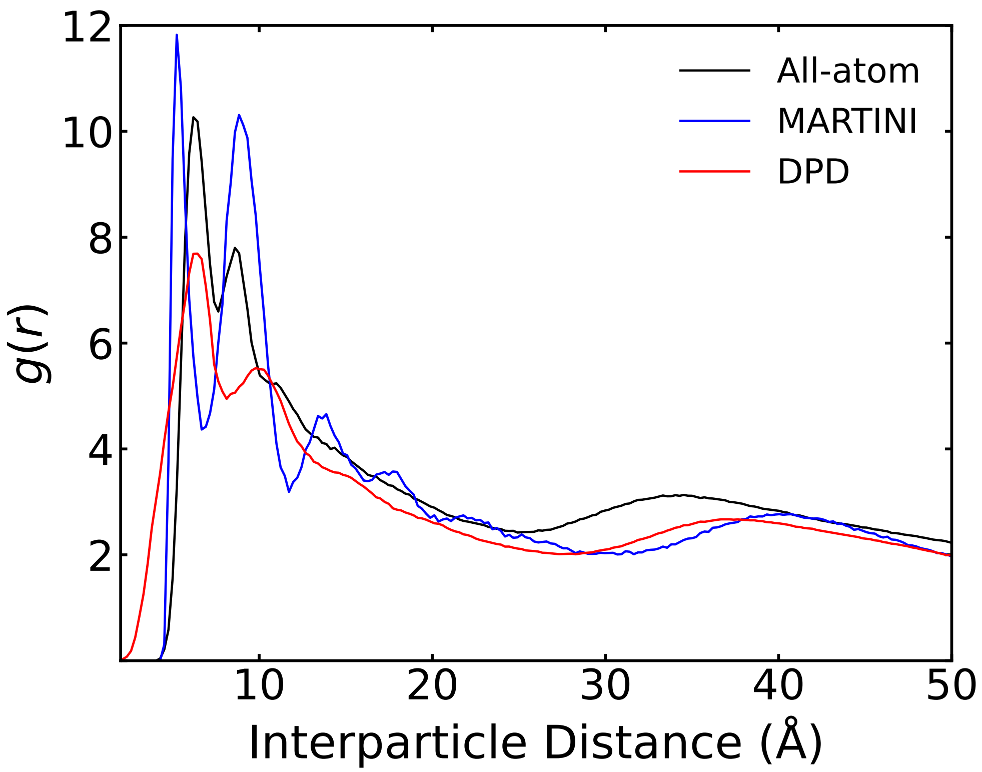

Figure S4: Phosphorus–phosphorus (P–P) radial distribution functions $g(r)$ of lipid head groups in a 12 nm vesicle, comparing all-atom, MARTINI, and DPD models. The phosphate P atom (all-atom) and PO4 bead (MARTINI and DPD) serve as the representative head group site for each model.

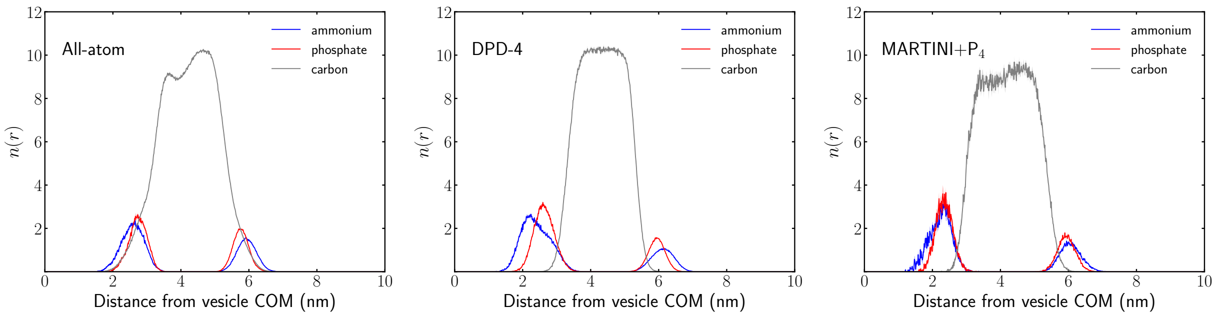

Figure S5: Block-averaged 12 nm density profiles for all-atom (left, 5 blocks of ∼100 frames), DPD-4 (center, 5 blocks of 100 frames), and MARTINI+P_4 (right, 5 blocks of ∼10 frames), with the shaded band showing the block-to-block standard error at each radial bin. Same data and binning convention as Fig. 1 of the main text. The shaded bands are narrow relative to the peak heights and may not be visible at first glance; zoom in on the curves to see them.

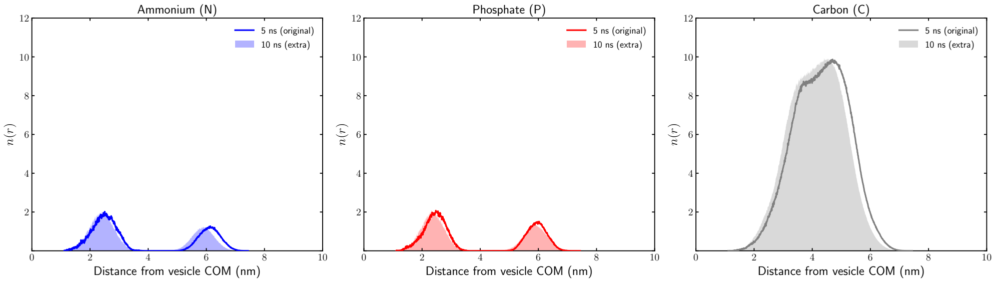

Figure S6: Radial distribution functions between the vesicle center of mass and left ammonium, center phosphate, and right carbon functional groups for the 12 nm all-atom DMPC vesicle, comparing the original 5 ns production run (solid line) to an independently extended 10 ns production run (shaded region) in each panel. The two distributions agree closely, confirming that the structural properties reported in the main text are converged with respect to simulation length.

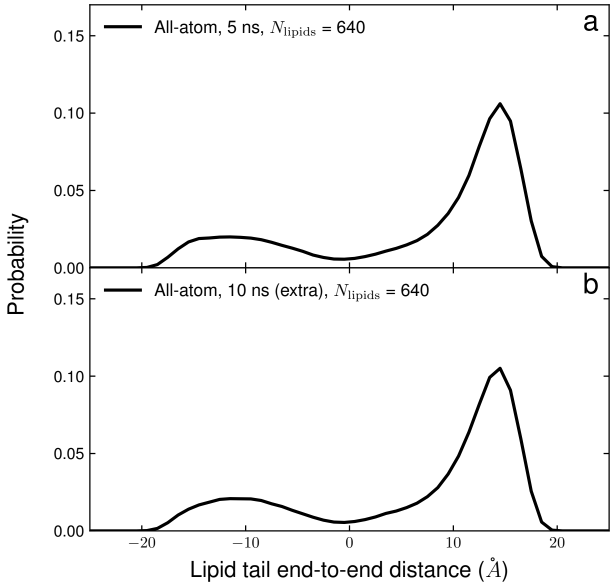

Figure S7: Distribution of lipid tail end-to-end distances, $r_{\rm ee}$, for the 12 nm all-atom DMPC vesicle from a) the original 5 ns production run and b) an independently extended 10 ns production run. The close agreement between the two distributions confirms that the lipid packing statistics reported in the main text are converged with respect to simulation length.

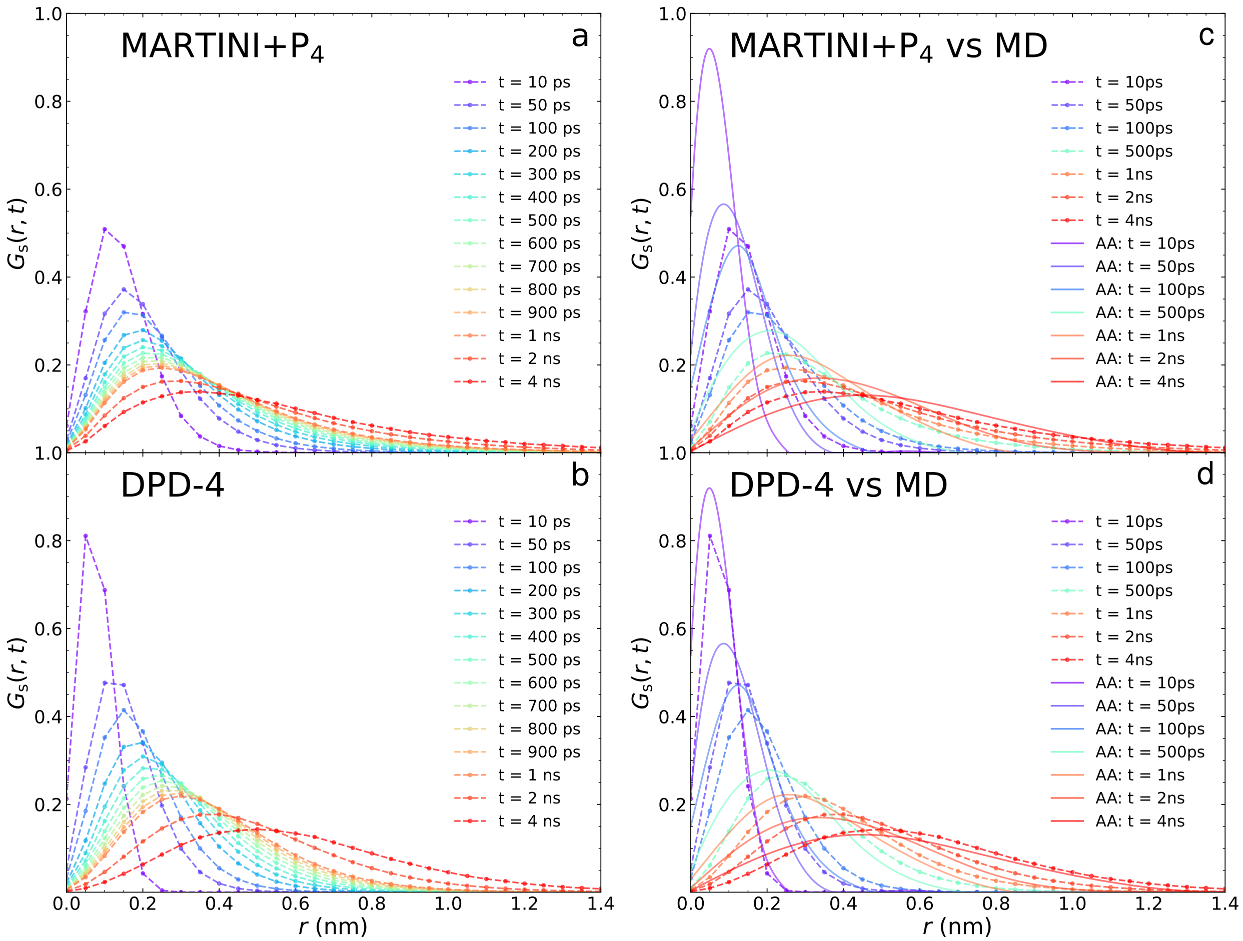

Figure S8: The van Hove function $G_s(r,t)$ for the lateral displacement r of lipids confined to the vesicle surface recorded at more times, t, than shown in Figure 5 of the main text. Here, $G_s(r,t)$ is the probability of a lipid moving a distance r relative to the surface over a fixed time interval t. Panels a and b present the van Hove functions obtained using MARTINI+P_4 and DPD-4, respectively, for the lipids on a nonplanar 60nm vesicle. They are reproduced in the corresponding panels c and d to their right as dashed curves, and superimposed with the MD generated values (solid curves) obtained for a planar surface as reported in Ref. 63.

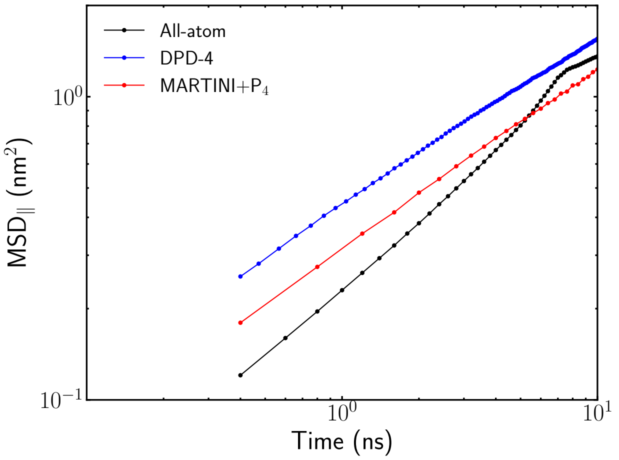

Figure S9: Mean-squared tangential (lateral) displacement of the phosphate head-group bead relative to the vesicle center of mass, for the 12 nm vesicle, on log–log axes. The local slope is the anomalous-diffusion exponent α (Table S5).

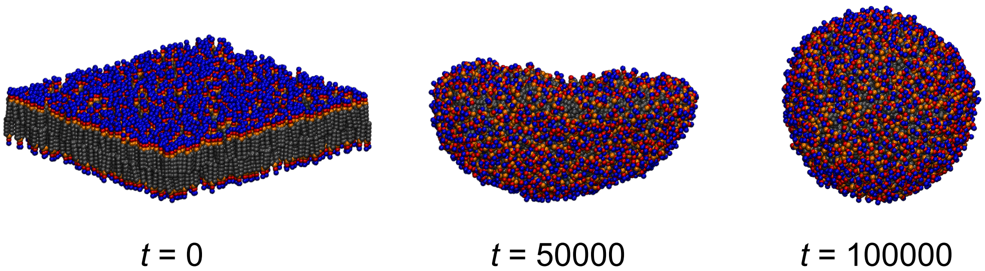

Figure S10: Vesicle self-assembly processes obtained using DPD-3 illustrates the formation of the vesicle in 100000 DPD time steps. The length scale is the same in all three figures, and can be inferred from the average thickness of the bilayer which is 4 nm as shown in the t=0 scheme.

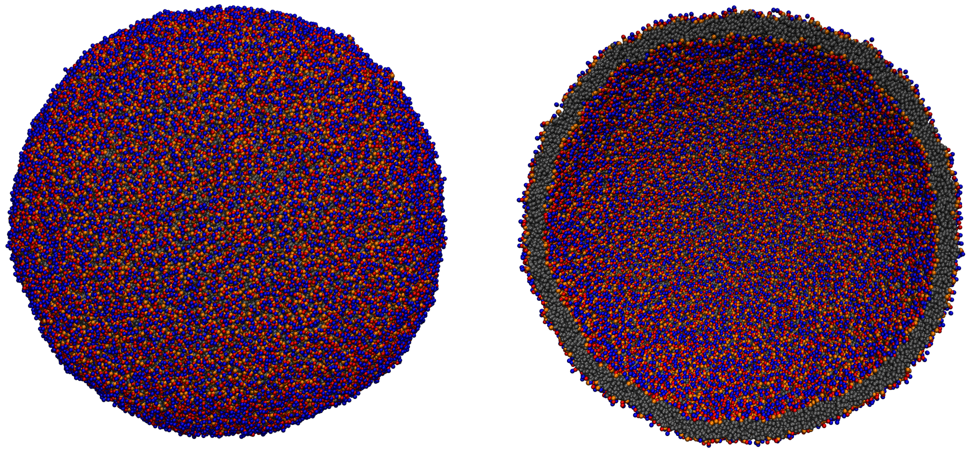

Figure S11: Representative structure of the 60 nm DPD-3 vesicle with 27848-lipid. The right image shows from the hemisphere to reveal the cross section along an equatorial plane, in support of the similar structures shown in Figure 4 in the main text for the DPD-4 and MARTINI vesicles. Ammonium head groups are in blue, phosphate head groups in orange, glycerol groups in red, and hydrophobic tail groups in gray for DPD-3 water beads are removed for clarity.

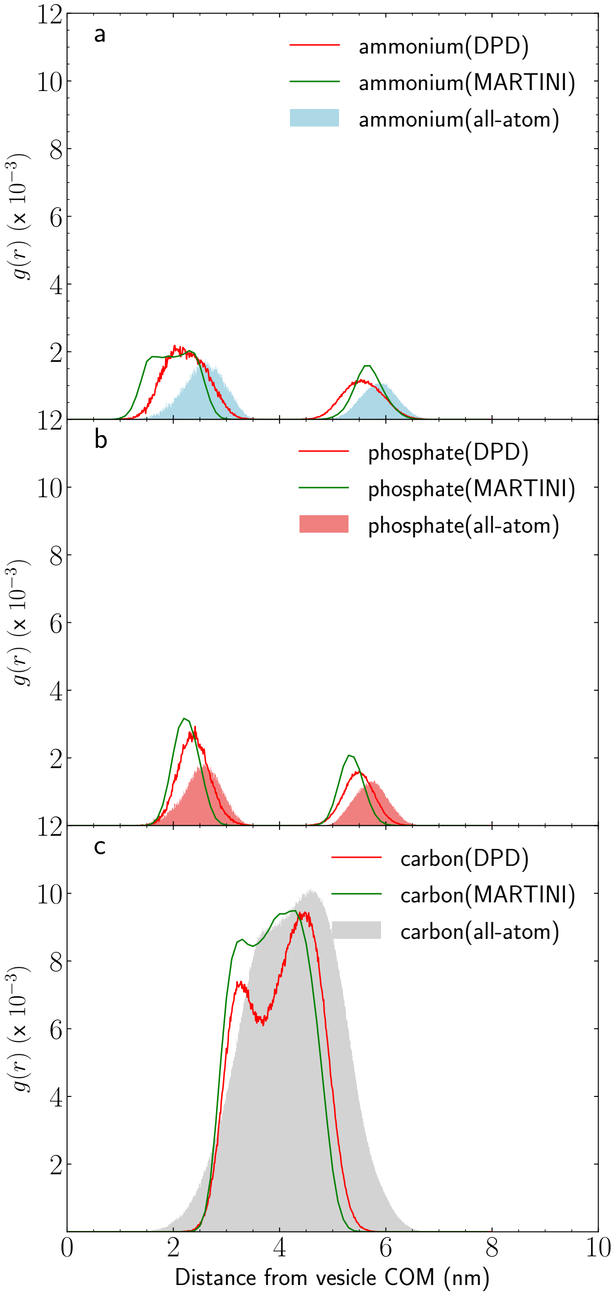

Figure S12: Distribution functions between different functional groups and the vesicle center of mass for the 12 nm vesicle using DPD-3, BMW-MARTINI, and all-atom models: a) ammonium functional group, b) phosphate functional group, and c) carbon atoms. This comparison supplements the distibutions shown in Figure 1 presented in the main text from all-atom, MARTINI-P4 and DPD-4 models.

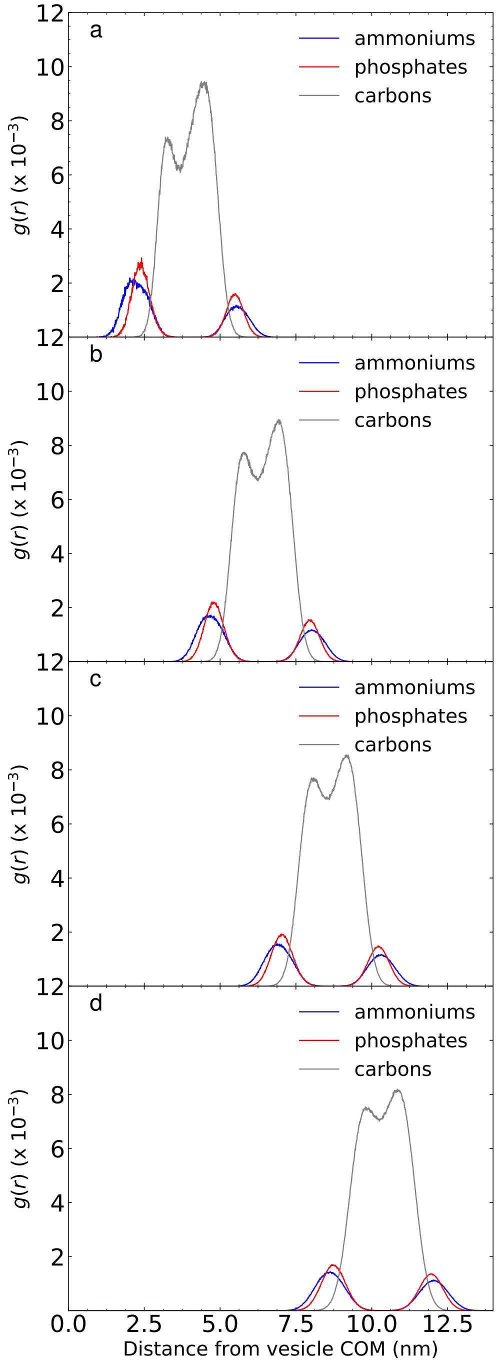

Figure S13: Radial distribution functions between lipid functional groups and the vesicle COM for vesicles of different sizes for DPD-3 models: a) ∼12-nm (576 lipids), b) ∼17-nm (1500 lipids), c) ∼22-nm (2750 lipids), and d) ∼25-nm (4000 lipids) in agreement with that obtained from DPD-4 models shown in Figure S3.

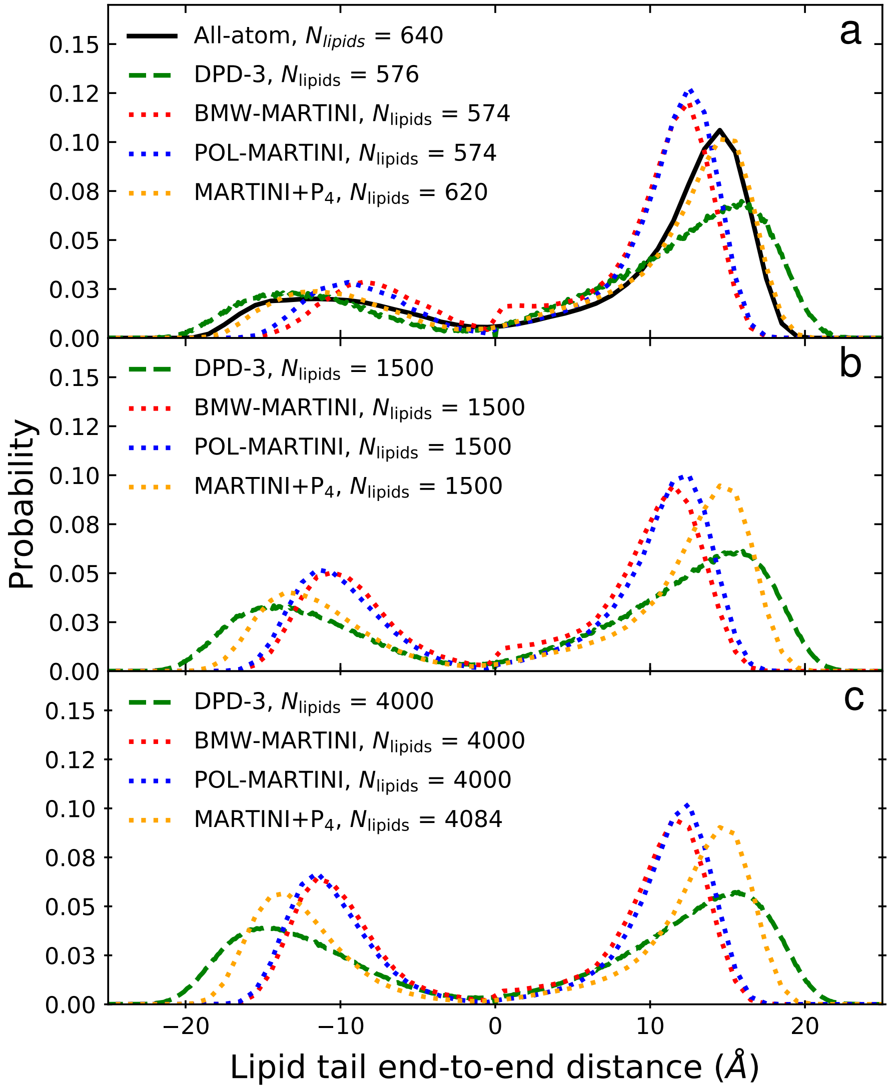

Figure S14: Distribution of lipid tail end-to-end distances, r_{ee}, in the inner (negative values) and outer (positive values) layers of a) 12 nm, b) 17 nm and c) 25 nm diameter DPD-3 model vesicles with 576, 1500 and 4000 total lipids. The DPD-3, BMW-MARTINI, POL-MARTINI, and MARTINI+P_4 models compared here complement the comparison between DPD-4 and MARTINI+P_4 shown in Figure 3 in the main text. In panel a, the r_{ee} distribution in all-atom DMPC-lipid vesicles are shown in black, along with representative DPD-3 small-r_{ee} splayed-tail and large-r_{ee} packed-tail lipid configurations. The distributions are normalized by the total number of frames sampled such that a frequency of 1.0 indicating one instance per frame. For comparison, the distributions for three MARTINI models are also shown in panels b and c.

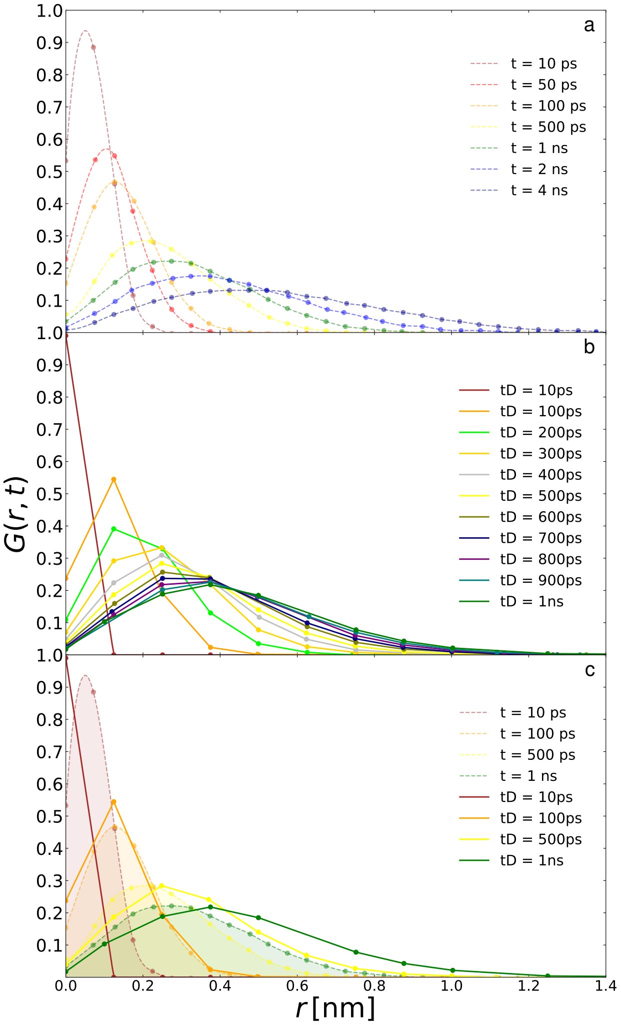

Figure S15: A comparison of the van Hove function $G_{\rm s}(r,t)$ for the lateral displacements of lipids obtained from a) all-atom MD[63] and b) DPD-3 simulations in support of the corresponding functions obtained from DPD-4and reported in Figure 5 in the main text. Here, $G_{\rm s}(r,t)$ is the probability of a lipid moving a distance r over a fixed time interval t. a) Reproduction of the values reported in Ref. 63 for the lipid COM generated using an all-atom representation of a 1152-PME system confined to a plane at the times indicated in the legend. b) Simulations for the 60nm Vesicle DPD-3 model wit references to the motion of the lipid tails on its near planar surface at the times indicated in the legend. c) Comparison between the two cases of panel a and panel b at various times that are in common between them.

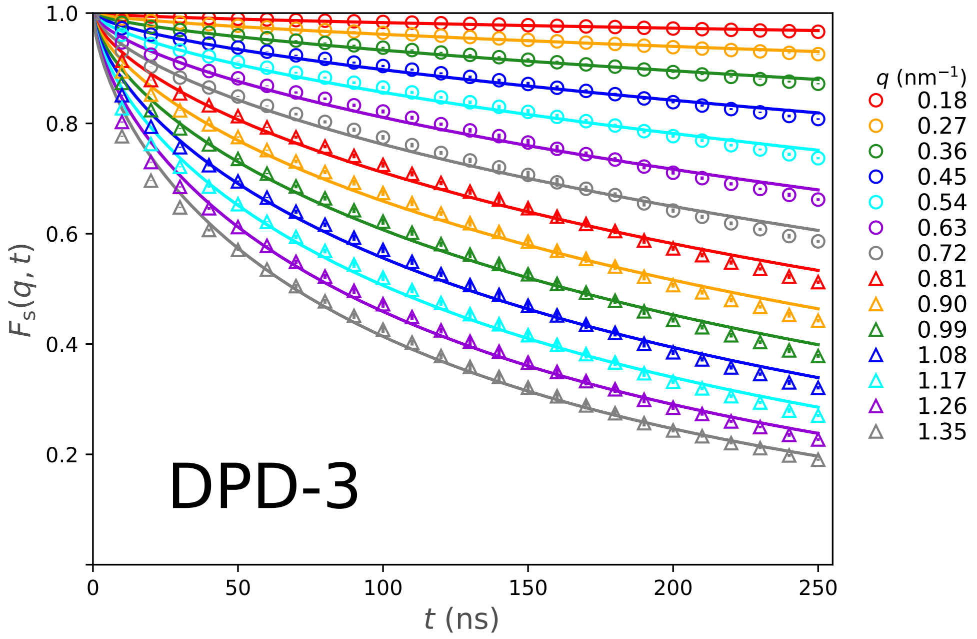

Figure S16: The intermediate scattering function $F_s(q,t)$ of a 60 nm vesicle from DPD-3 simulations (markers) with error bars within markers and fits from the Zilman-Granek model (solid lines) over a range of wave numbers q in support of that obtained from DPD-4 and reported in Figure 6 in the main text.

References

Supercited reference numbers in this SI refer to the reference list of the main paper.
